# Graphene oxide quantum dots-loaded sinomenine hydrochloride nanocomplexes for effective treatment of rheumatoid arthritis via inducing macrophage repolarization and arresting abnormal proliferation of fibroblast-like synoviocytes

**DOI:** 10.1186/s12951-024-02645-8

**Published:** 2024-07-01

**Authors:** Ye Lin, Yuanyuan Tang, Ouyang Yi, Junping Zhu, Zhaoli Su, Gejing Li, Hua Zhou, Liang Liu, Bin Liu, Xiong Cai

**Affiliations:** 1 Institute of Innovation and Applied Research in Chinese Medicine, Department of Rheumatology of First Hospital, Hunan University of Chinese Medicine, Changsha, 410208 Hunan China; 2 College of Biology, Hunan University, Changsha, 410082 Hunan China; 3 State Key Laboratory of Traditional Chinese Medicine Syndrome, The Second Affiliated Hospital of Guangzhou, Guangdong Provincial Hospital of Chinese Medicine, Guangdong Provincial Academy of Chinese Medical Sciences, University of Chinese Medicine, Guangzhou, 510006 China

**Keywords:** Sinomenine hydrochloride, Nanomedicine, Rheumatoid arthritis, Fibroblast-like synoviocytes, Macrophage repolarization

## Abstract

The characteristic features of the rheumatoid arthritis (RA) microenvironment are synovial inflammation and hyperplasia. Therefore, there is a growing interest in developing a suitable therapeutic strategy for RA that targets the synovial macrophages and fibroblast-like synoviocytes (FLSs). In this study, we used graphene oxide quantum dots (GOQDs) for loading anti-arthritic sinomenine hydrochloride (SIN). By combining with hyaluronic acid (HA)-inserted hybrid membrane (RFM), we successfully constructed a new nanodrug system named HA@RFM@GP@SIN NPs for target therapy of inflammatory articular lesions. Mechanistic studies showed that this nanomedicine system was effective against RA by facilitating the transition of M1 to M2 macrophages and inhibiting the abnormal proliferation of FLSs in vitro. In vivo therapeutic potential investigation demonstrated its effects on macrophage polarization and synovial hyperplasia, ultimately preventing cartilage destruction and bone erosion in the preclinical models of adjuvant-induced arthritis and collagen-induced arthritis in rats. Metabolomics indicated that the anti-arthritic effects of HA@RFM@GP@SIN NPs were mainly associated with the regulation of steroid hormone biosynthesis, ovarian steroidogenesis, tryptophan metabolism, and tyrosine metabolism. More notably, transcriptomic analyses revealed that HA@RFM@GP@SIN NPs suppressed the cell cycle pathway while inducing the cell apoptosis pathway. Furthermore, protein validation revealed that HA@RFM@GP@SIN NPs disrupted the excessive growth of RAFLS by interfering with the PI3K/Akt/SGK/FoxO signaling cascade, resulting in a decline in cyclin B1 expression and the arrest of the G2 phase. Additionally, considering the favorable biocompatibility and biosafety, these multifunctional nanoparticles offer a promising therapeutic approach for patients with RA.

**Graphical abstract:**

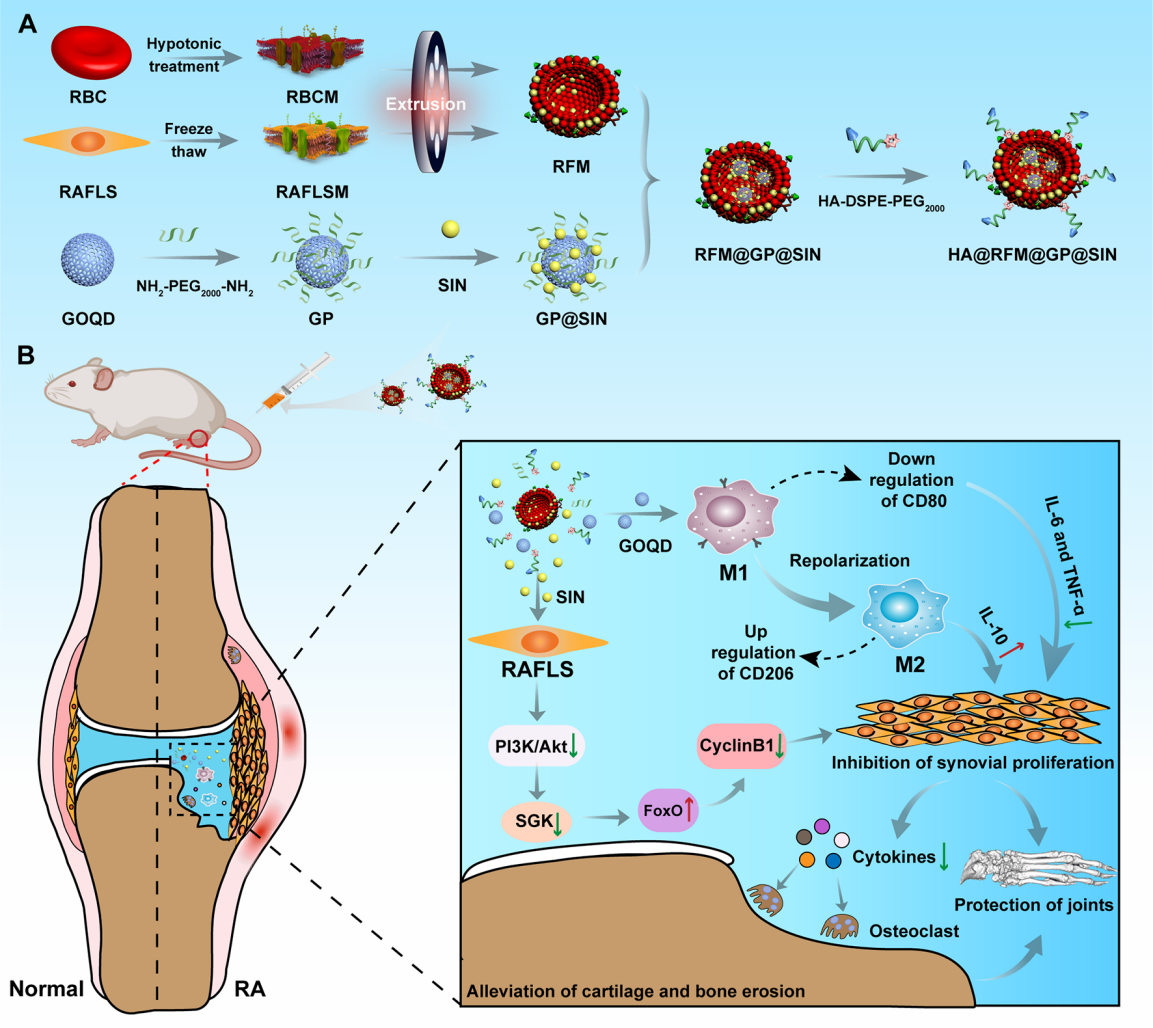

**Supplementary Information:**

The online version contains supplementary material available at 10.1186/s12951-024-02645-8.

## Introduction

Rheumatoid arthritis (RA) is an intricate immune-mediated disease characterized by synovial inflammation and hyperplasia, leading to progressive joint destruction [1, 2]. In RA, the interactions between fibroblast-like synoviocytes (FLSs) with macrophages have emerged as crucial contributors to joint damage [3, 4]. FLSs in RA can attract macrophages from the vasculature to the synovium through the secretion of chemotactic factors. These macrophages are the primary sources of critical pro-inflammatory cytokines, which further promote the rapid proliferation and division of FLSs, thus exacerbating RA [5, 6]. Consequently, a promising strategy for the treatment of RA involves simultaneous regulation of macrophage repolarization to alleviate inflammation and the inhibition of abnormal FLS proliferation.

Sinomenine, an active compound derived from the traditional Chinese medicinal plant *Sinomenium acutum*, part of the Menispermaceae family, showed strong anti-rheumatic property [7]. Presently, sinomenine hydrochloride (SIN) formulations, marketed under the name Zhengqing Fengtong Ning, are employed in treating RA and similar rheumatic diseases. These formulations are designed to curb the growth, invasive activity, and movement of FLSs [8, 9]. Nonetheless, SIN’s rapid metabolism and brief half-life might provoke adverse reactions with high-dose and long-term use [10]. Consequently, there’s a pressing demand for new approaches to enhance SIN’s efficacy while reducing potential side effects [11].

Recently, many advanced nanosystems have been adopted to enhance drug concentration at the site of lesion and minimize side effects in the treatment of RA [12, 13]. Among them, metal-organic frameworks [14] and synthetic polymers [15] have been developed for delivering SIN. Despite the potential benefits of these designs, practical implementation is hindered by several persistent issues, such as the lack of a multifunctional collaborative approach, suboptimal therapeutic effects, and concerns regarding the biosafety. As a new 2-deminsional nanomaterial, graphene oxide quantum dots (GOQDs) have gained attention for drug delivery due to their non-toxic nature, versatile bioactivity, and biodegradability [16, 17]. Moreover, GOQDs have demonstrated the anti-inflammation ability to promote the repolarization of pro-inflammatory macrophages (M1) into anti-inflammatory macrophages (M2), which is extremely beneficial for the treatment of autoimmune disorders [18]. Thus, GOQDs not only function as carriers but also facilitate the transformation of macrophages from the M1 to M2 phenotype to exert antioxidant and anti-inflammatory effects, enabling collaborative therapy. This motivates us to employ GOQDs as drug and a safe carrier to deliver SIN, specifically targeting macrophages and FLSs, to achieve synergistic treatment for RA.

Here, utilizing the small particle size of GOQD-NH_2_-PEG_2000_-NH_2_ nanoparticles (GP NPs) and high drug loading capacity and biosafety, we developed a multifunctional nanodrug delivery system of GP@SIN NPs. These NPs were designed for the polarization of inflammatory macrophages and the inhibition of FLS proliferation. Additionally, we fabricated a hybrid membrane (RFM) coating composed of red blood cell membranes (RBCM) and RAFLS membranes (RAFLSM) to confer biomimetic properties to GP@SIN, enhancing immune evasion and homologous targeting capabilities [19]. Furthermore, hyaluronic acid (HA) modification was implemented to improve the uptake efficiency of HA@RFM@GP@SIN NPs by FLSs and activated macrophages at the arthritic sites [20]. This nanodrug delivery system demonstrated precise targeting of the synovial lesion site in arthritis, synergistic regulation of macrophage polarization and synovial hyperplasia, and efficient prevention of cartilage erosion and bone destruction.

## Materials and methods

### Cell

Mycoplasma-free cell lines, including human FLS (HFLS), RAFLS, human liver cells (HL7702), RAW264.7 cells (mouse macrophages), human umbilical vein endothelial cells (HUVEC), rat myocardial cells (H9C2), vascular smooth muscle cells (VSMC), and human kidney-2 (HK2), were procured from the Cell Bank of the Chinese Academy of Sciences (Beijing, China). These cell types were maintained in Dulbecco’s Modified Eagle Medium (Thermo Fisher Scientific), enriched with 10% fetal bovine serum (FBS) and 1% penicillin and streptomycin (Solarbio Life Sciences, Beijing, China), and cultivated at 37℃ in a 5% CO_2_ atmosphere.

### Animals

Sprague-Dawley (SD) rats (Certificate of Quality nos. ZS-202,206,140,002, ZS-202,203,150,022) were obtained from Guangzhou Vital River Laboratory Animal Technology Company (Guangzhou, China). This study involved 4-week-old male rats weighing 90–110 g and 5-week-old female rats weighing 130–150 g. These rats were accommodated at the Laboratory Animal Center affiliated with the Hunan University of Chinese Medicine (HNUCM) (License No. SYXK [Hunan] 2019-0009), ensuring continuous access to feed and water. The HNUCM’s Institutional Animal Care and Use Committee sanctioned all experimental designs and animal maintenance protocols, ensuring they aligned with the National Institute of Health’s guidelines for the humane care and handling of laboratory animals.

### Preparation of GP@SIN NPs

GP NPs were prepared following a previously established procedure [21]. In brief, GOQDs (Nanjing XENANO Materials Tech Co., Ltd, Nanjing, China), 1-ethyl-3-(3-dimethyl aminopropyl) carbodiimide (EDC) (Sigma-Aldrich Corp., St. Louis, MO, USA), and N-hydroxy-succinimide (NHS) (Sigma-Aldrich) were combined at 1:40:10 and stirred magnetically for 30 min. Next, NH_2_-PEG_2000_-NH_2_ (Ponsure Inc., Shanghai, China) was supplemented and magnetically stirred for 24 h. Dialysis was performed to eliminate any unencapsulated NH_2_-PEG_2000_-NH_2_. Subsequently, the purified GP NPs were lyophilized and preserved at -80 °C for future use.

GP@SIN NPs: GP NPs were loaded with SIN (Hunan Zhengqing Pharmaceutical Group Co. LTD, Huaihua, Hunan, China) through electrostatic adsorption interactions. To achieve this, SIN and GP NPs were dissolved together in water at a mass ratio of 3:1 (w/w) and stirred magnetically for 24 h. Dialysis was then performed to remove any unencapsulated SIN from the solution. Finally, the purified GP@SIN NPs were lyophilized and preserved at -80 °C for future use.

The quantification of SIN in GP@SIN NPs was completed utilizing the ultraviolet-visible (UV-vis) absorption spectrum (Beckman Coulter, Brea, CA, USA). To accomplish this, the GP@SIN solution was placed in a dialysis bag (WMCO = 3500 D) and submerged in water. The concentration of unencapsulated SIN in the solution outside the dialysis bag was determined at a wavelength of 262 nm. The drug entrapment efficiency (DEE) and drug loading efficiency (DLE) of SIN were calculated utilizing the subsequent equations.

1 $${\text{DEE}}\,{\text{(\%)}} = \frac{{\left( {{\text{Ms - Mu}}} \right)}}{{{\text{Ms}}}} \times100\%$$

2 $${\text{DLE}}\,{\text{(\% )}} = \frac{{{\text{(Ms - Mu)}}}}{{{\text{Mg}}}}\,{ \times 100\% }$$

Where Ms, Mu, and Mg denote the masses of added SIN, unencapsulated SIN, and GP, respectively.

### RBCM and RAFLSM preparation

The RBCM and RAFLSM were extracted according to the previous report [22]. Briefly, whole blood samples were harvested from the abdominal aorta of normal adult SD rats, followed by centrifugation at 3500 rpm and 4℃ for 10 min. The resulting precipitate consisted of erythrocytes. These erythrocytes were then rinsed with 1×phosphate buffered saline (PBS). To hemolyze the red blood cells (RBCs), a hypotonic solution (0.25×PBS) was utilized. Purified RBCM was obtained by repeatedly cleaning the hemolysates with 0.25×PBS.

The rat RAFLS cells were prepared using Membrane Protein Reagent A (Beyotime Biotechnology Co., Ltd., Shanghai, China), with added phenylmethylsulfonyl fluoride, and incubated on ice for 1 h. The mixture was first centrifuged at 800 rpm for 10 min at 4 °C to separate the supernatant, which was then further centrifuged at 12,500 rpm for 30 min at the same temperature to isolate the pure membrane fraction. Protein concentrations of both RBCM and RAFLSM were determined utilizing the BCA Protein Assay Kit (Solarbio).

### Preparation of HA-DSPE-PEG_2000_-NH_2_

HA-DSPE-PEG_2000_-NH_2_ was prepared following a previously established procedure [23]. Briefly, HA (Meilune Biotechnology Co., Ltd, Dalian, China), EDC, and NHS were combined in PBS at a weight ratio of 1:5:10 and subjected to magnetic stirring for 30 min. Afterward, DSPE-PEG_2000_-NH_2_ (Ponsure Inc., Shanghai, China) was incorporated into the mixture, with stirring extended for an additional 24 h. Dialysis was employed to remove any unbound DSPE-PEG_2000_-NH_2_ from the HA-DSPE-PEG_2000_-NH_2_ formulation. The purified HA-DSPE-PEG_2000_-NH_2_ was then lyophilized and preserved at 4 °C for subsequent use.

### Synthesis of HA@RFM@GP@SIN NPs

The RFM hybrid, combining RAFLSM and RBCM in equal volumes at a concentration of 1 mg/mL, was synthesized. This was followed by blending 1 mL of RFM with 1.25 mg of GP@SIN NPs, and the concoction was agitated at 37 °C for 2 h. The solution containing RFM and GP@SIN NPs was squeezed at least 10 times through the micro-extruder before centrifugation at 3000 rpm for 30 min. After discarding the precipitation (uncoated membrane), the supernatant was centrifuged at 13,000 rpm for 10 min to obtain RFM@GP@SIN (precipitation). Subsequently, the mixture was enhanced by adding 0.625 mg of HA-DSPE-PEG_2000_-NH_2_ dissolved in PBS, and continuous stirring was maintained for 1 h. This procedure yielded the HA@RFM@GP@SIN NPs, achieving concentrations of 1.11 mg/mL for either GP NPs or SIN.

### Membrane hybridization study

RBCM and RAFLSM were respectively stained with DiI and DiO dyes, utilizing kits provided by Yeasen Biotechnologies Co., Ltd (Shanghai, China). Subsequently, the hybridized RFM was generated by combining equal volumes of labeled RBCM and RAFLSM at a concentration of 1 mg/mL. The hybridization of RAFLSM-RBCM within RFM was then examined and imaged via a confocal laser scanning microscope (CLSM) (Olympus, Tokyo, Japan).

### Characterization of membrane proteins

Membrane protein specimens were fractionated employing 10% sodium dodecyl sulfate-polyacrylamide gel electrophoresis (SDS-PAGE) until bromophenol blue reached the bottom. Post-separation, these proteins were transferred onto a polyvinylidene fluoride membrane. To obtain the total protein map, the gel was stained with Coomassie bright blue. The membrane was subsequently utilized for the characterization of specific surface markers of RAFLSM and RBCM, such as CD44 and CD47. Additionally, a UV-vis spectrophotometer was utilized for identifying unique absorbance peaks of RBCM, RAFLSM, and RFM in the protein samples within the 200–600 nm range.

### Specific targeting of RFM

GP NPs were labeled with rhodamine (Rho). HFLS and RAFLS cells (7 × 10^4^ cells/well) were seeded in 12-well plates for 24 h. Cells were then incubated in fresh medium containing RBCM@GP^Rho^ NPs, RAFLSM@GP^Rho^ NPs, or RFM@GP^Rho^ NPs (40 µg/mL GP NPs) for 4 h to investigate the specific targeting of RFM. The nuclei of the cells were then stained utilizing Hoechst 33,342 (Yeasen Biotechnologies Co., Ltd., Shanghai, China). Imaging was performed employing a CLSM.

### Characterization of HA@RFM@GP@SIN NPs

The morphology of HA@RFM@GP@SIN NPs was examined utilizing transmission electron microscopy (TEM) (Japan Electron Optics Laboratory, Tokyo, Japan). Measurements of the NP sizes and their zeta potential were implemented utilizing a Zetasizer Nano ZSP (Malvern, UK). The presence of functional groups in HA@RFM@GP@SIN NPs was identified utilizing a Fourier-transform infrared spectrometer (FT-IR).

### HA@RFM@GP@SIN NPs uptake assay in vitro

HFLS, RAFLS, RAW264.7, and lipopolysaccharide (LPS)-stimulated RAW264.7 cells were employed to assess their capacity for cellular uptake. These cells (7 × 10^4^ cells/well) were seeded into 12-well plates and allowed to adhere for 24 h. GP NPs were labeled with Rho. Subsequently, these were administered with either RFM@GP@SIN NPs or HA@RFM@GP@SIN NPs (40 µg/mL, GP) for 4 h. For nanoparticle targeting evaluation, cells underwent pre-treatment with 500 µg/mL of HA for 1 h, followed by exposure to HA@RFM@GP@SIN NPs for an additional 4 h. Hoechst 33,342 dye was utilized for nuclear staining of the cells, which were subsequently analyzed utilizing a CLSM.

### In vitro cellular uptake mechanism

The mechanism of uptake for HA@RFM@GP@SIN NPs was investigated by performing preincubation of RAFLS with specific uptake inhibitors, followed by incubation with the nanomaterials [14]. The nanomaterials were labeled with Rho. In brief, RAFLS was incubated for 1 h with specific inhibitors - methyl-β-cyclodextrin (M-β-CD, caveolae-mediated endocytosis inhibitor) (Solarbio), chlorpromazine (Chlor, clathrin-dependent endocytosis inhibitor) (Sigma-Aldrich), and colchicine (Colch, micropinocytosis inhibitor) (Solarbio). After that, the culture medium was removed, and HA@RFM@GP@SIN NPs (40 µg/mL, GP) were added and incubated for 4 h. Hoechst 33,342 dye was utilized to stain the cell nucleus. The cells were then observed using CLSM.

### Subcellular localization of HA@RFM@GP@SIN NPs

To detect the subcellular localization of HA@RFM@GP@SIN NPs, the nanomaterial was labeled with Rho. RAFLS cells were cultivated at a density of 7 × 10^4^ cells per well for 24 h. Subsequently, the cells underwent incubation with HA@RFM@GP@SIN NPs (40 µg/mL, GP) for varying durations of 0, 2, 4, 6, and 8 h. After rinsing with PBS, the organelles were stained with 1 µM Lysosome Tracker Red DND-99 (lysosomes) (Yeasen Biotechnologies Co., Ltd., Shanghai, China) for 30 min. Nuclei of the cells were marked using Hoechst 33,342 dye. Ultimately, the cells were examined with a CLSM.

### Regulating the M1 to M2 phenotypic transition of macrophages in vitro

RAW264.7 cells (2 × 10^5^ cells/well) were cultured in 24-well plates, with some groups exposed to 500 ng/mL LPS for 24 h. Subsequently, cells were treated with PBS, SIN (100 µM), GP NPs (40 µg/mL), or HA@RFM@GP@SIN NPs (100 µM SIN and 40 µg/mL GP) for 48 h. Post-incubation, cells were fixed with 4% paraformaldehyde, permeabilized with 0.3% Triton X-100, and blocked with 5% BSA to reduce non-specific binding. They were then incubated with anti-CD80 or anti-CD206 antibodies overnight at 4 °C. Secondary antibody staining was executed with Dylight-594 or Dylight-488 labeled antibodies (Proteintech Group Inc., Rosemont, IL, USA) for 2 h at ambient temperature. Nuclei were then stained utilizing DAPI for 10 min before microscopic examination employing a Nikon fluorescence microscope. The ImageJ software was used to assess the mean fluorescence intensity (MFI) of images.

In parallel, RAW264.7 cells (1 × 10^6^ cells/well) were cultured in 6-well plates under similar conditions. Post-LPS and NP treatments for 48 h, cells were stained with BD Horizon™ Fixable Viability Stain, followed by surface marker blocking with BD Fc Block and staining with PE-conjugated CD80 antibody (Biolegend Inc., USA) for 30 min at 4 °C. Following the surface staining, cells were fixed, permeabilized utilizing the BD Pharmingen™ Transcription Factor Buffer Set, and stained intracellularly with APC-conjugated CD206 antibody (Biolegend Inc., USA) for 30 min at 4 °C. Analysis was performed employing a BD LSRFortessa flow cytometer, with FlowJo Software for data interpretation.

RAW264.7 cells were cultured for 24 h in a 6-well plate, then treated with PBS, SIN, GP NPs, or HA@RFM@GP@SIN NPs for 2 h, followed by incubation with 500 ng/mL LPS for 4 h. Cells were then stained with 2’,7’-Dichlorodihydrofluorescein diacetate (DCFH-DA, Yeasen Biotech, Shanghai, China) and analyzed utilizing flow cytometry.

RAW264.7 cells in 24-well plates were incubated with PBS, SIN, GP NPs, or HA@RFM@GP@SIN NPs for 2 h and then co-incubated with 100 ng/mL LPS for an additional 48 h. After that, the culture media were collected, and the levels of tumor necrosis factor-α (TNF-α), interleukin-6 (IL-6), and interleukin-10 (IL-10) were measured utilizing an Enzyme-Linked Immunosorbent Assay (ELISA, Neobioscience Biotechnology, Shenzhen, China) kit.

### Targeting RAFLS growth arrest of HA@RFM@GP@SIN NPs in vitro

The impact of nanodrugs on the viability of RAFLS was assessed by performing an MTT assay (Solarbio). RAFLS cells were plated at a concentration of 7 × 10^3^ cells per well in 96-well plates and incubated for 24 h. Subsequently, fresh media containing either PBS, SIN (100 µM), GP NPs (40 µg/mL), or HA@RFM@GP@SIN NPs (100 µM SIN and 40 µg/mL GP) was added, and the cells were cultured for an additional 48 h. The cytotoxicity of the nanodrugs was evaluated utilizing the MTT assay.

Additionally, RAFLS cells were plated in 12-well plates (7 × 10^4^ cells/well) for a 24h of incubation. Following the above treatment, the cells were treated with calcein-AM solution at 37 °C for 30 min (dark condition). The fluorescence of the RAFLS cells was subsequently captured and evaluated employing a CLSM. For cell proliferation assessment, 5-Ethynyl-2’-deoxyuridine (EdU) was incorporated into the culture 4 h before concluding the experiment. The cells were then stained utilizing an EdU assay kit (Beyotime), and the proliferation rate was assessed utilizing a CLSM.

RAFLS cells were plated in 96-well plates (7 × 10^3^ cells/well) for 24 h of incubation. Following the same treatment as above for 0, 1, 2, 3, 4, 5, 6, and 7 days, RAFLS cell viability was tested utilizing the MTT assay. The rate of cell growth was determined by applying the formula:

Cell growth rate = OD490 nm (0, 1, 2, 3, 4, 5, 6 or 7 days)/ OD490 nm at Day 0).

To profile the cell cycle, RAFLS cells were seeded in 6-well plates (1.5 × 10^5^ cells/well). Following the same treatment as above for 24 h, the cells were harvested and processed using a Cell Cycle Detection Kit (Solarbio). Cell cycle analysis was completed with the help of a flow cytometer (Beckman Coulter, Brea, CA, USA).

Western blotting (WB) was implemented to evaluate protein expression in cells. In summary, RAFLS cells were seeded in 6-well plates (1.5 × 10^5^ cells/well) and subjected to treatment with either PBS, SIN (100 µM), GP NPs (40 µg/mL), or HA@RFM@GP@SIN NPs (100 µM SIN and 40 µg/mL GP). After 48 h of incubation, cells were lysed with RIPA buffer for total protein extraction. The expression of cyclin B1 and p53 (Proteintech) was then determined utilizing WB.

RAFLS cells (1.5 × 10^5^ cells/well) were seeded into 6-well plates and incubated at 37 °C with 5% CO_2_ for 24 h. Following the same treatment as above for 48 h, cell staining was implemented utilizing the Annexin V-FITC/PI Apoptosis Detection Kit (Yeasen Biotechnologies Co., Ltd., Shanghai, China). Subsequent analysis was conducted utilizing flow cytometry.

### In vivo pharmacokinetic study and targeting of HA@RFM@GP@SIN NPs

On day 36 after injection of Complete Freund’s adjuvant (CFA), rats with adjuvant-induced arthritis (AIA) were intravenously administered Chlorin e6 (Ce6), GP^Ce6^ NPs, or HA@RFM@GP@SIN^Ce6^ NPs at a dose of 5 mg/kg Ce6. Subsequent to administration, blood samples of 0.3 mL were extracted from the jugular veins at various time intervals: immediately (0), 0.5, 1.5, 3, 6, 12, 24, and 48 h post-injection. The plasma pharmacokinetics of HA@RFM@GP@SIN NPs were then evaluated utilizing an IVIS Kinetics optical system (PerkinElmer, USA). The paw distribution of the drug was evaluated at 0.5, 3, 6, 12, 24, and 48 h using the same system. After 48 h, tissue samples from the heart, lung, spleen, liver, kidney, and paw were imaged and analyzed for drug biodistribution.

### Therapeutic efficacy of HA@RFM@GP@SIN NPs in AIA rats

The AIA rat model was employed to assess the significance of HA@RFM@GP@SIN NPs on RA. Initially, a subcutaneous injection of 0.1 mL of CFA was administered to male SD rats to induce AIA. Starting from day 0, rats were intravenously injected with PBS, SIN (6 mg/kg), GP NPs (6 mg/kg), or HA@RFM@GP@SIN NPs (6 mg/kg SIN and 6 mg/kg GP) every three days into the tail until day 36. The normal rats received PBS injections. In the positive control group, AIA rats were orally administered methotrexate (MTX, 1 mg/kg) on days 0, 3, 7, 14, 21, 28, and 35 following CFA injection. Evaluations included the severity of arthritis, bi-hind paw volume measurements, and body weight tracking, conducted tri-weekly following the onset of arthritis.

Bone erosion in rats was assessed based on radiographs, with each paw being scored on a 0–4 scale [14, 22]. A score of 0 represented good correspondence between the interphalangeal joint and metatarsophalangeal joint, normal articular cavity, and no marginal osteophytes with a smooth articular surface. A score of 1 indicated a slightly narrowed articular cavity, less marginal osteophytes, and slight roughness in the articular surface. A score of 2 denoted low-to-moderate narrowing of the articular cavity, low-to-moderate presence of marginal osteophytes, and low-to-moderate roughness in the articular surface. A score of 3 represented a narrowed articular cavity, pronounced presence of marginal osteophytes, and pronounced roughness in the articular surface. A score of 4 indicated extremely poor or no correspondence between the interphalangeal joint and metatarsophalangeal joint, significant presence of marginal osteophytes, and severe roughness in the articular surface. Following the experiment, the rats were euthanized through isoflurane asphyxiation. Hind paws and blood samples were collected. The hind paws were fixed in 4% paraformaldehyde (Solarbio) and decalcified using ethylenediaminetetraacetic acid (EDTA) decalcifying solution (Solarbio) before paraffin embedding, followed by hematoxylin and eosin (H&E) and Safranin O staining. Histopathological synovial scores (HSS) were then evaluated by two independent observers using H&E-stained sections of the ankle joints. The scoring was done on a semiquantitative four-point scale ranging from 0 to 4, with 0 representing normal, and 1, 2, 3, and 4 denoting mild, moderate, severe, and extremely severe changes, respectively. Safranin O staining sections of the ankle joint were used for the Mankin scores assessment [24], which evaluated the cartilage structure, chondrocytes, cartilage matrix, and the integrity of the tidal line. The tissue immunofluorescence staining was performed to evaluate the M1 to M2 phenotypic transition of macrophages. To examine synovium inflammation, ankle synovium sections were stained with anti-IL-10, anti-TNF-α (Proteintech), and anti-IL-6 antibodies (Boster Biological Technology, Ltd., Wuhan, China). The immunohistochemical staining results were visualized utilizing an Olympus FV1200 microscope (Olympus, Tokyo, Japan). Quantitative analysis was performed using ImageJ software. Serum levels of IL-10, TNF-α, and IL-6 cytokines were tested employing ELISA kits (Neobioscience Biotechnology, Shenzhen, China).

### Anti-arthritic effects and metabolomics analysis of HA@RFM@GP@SIN NPs in collagen-induced arthritis (CIA) rats

CIA was initiated in female SD rats employing an emulsion of Bovine Type-II Collagen and Incomplete Freund’s Adjuvant (Chondrex, Inc., Woodinville, WA, USA). The rats were first injected subcutaneously with 0.2 mL of this emulsion and received a booster shot after seven days. From day 0, the CIA rats were treated intravenously every three days with either PBS or HA@RFM@GP@SIN NPs (6 mg/kg each of SIN and GP) until day 36, while normal control rats received PBS. The positive control group was given oral MTX (1 mg/kg) on days 0, 3, 7, 14, 21, 28, and 35. Paw volume and arthritis scores were evaluated every three days, and all rats were euthanized on day 36. Plasma samples from different groups were analyzed using LC-MS by Novogene, Beijing.

### RNA-seq analysis and WB verification

The RAFLS cells were treated with PBS or HA@RFM@GP@SIN NPs for 48 h. RNA samples were harvested from both the control and HA@RFM@GP@SIN groups (*n* = 3) for RNA-seq analysis, which was conducted by BioMarker (Beijing, China).

RAFLS cells (1.5 × 10^5^ cells/well) were seeded into 6-well plates and treated with PBS or HA@RFM@GP@SIN NPs (100 µM SIN and 40 µg/mL GP). After 48 h of culture, cells were harvested and lysed using RIPA buffer. Primary antibodies targeting PI3K (1:1000), p-PI3K (1:1000), Akt (1:4000), p-Akt (1:1000), SGK2 (1:1000), FoxO4 (1:1000), Cyclin B1 (1:5000), and β-actin (1:20000) (Cell Signaling Technology, Inc. and Proteintech) were used. Protein levels were measured using chemiluminescence after incubation with secondary antibodies, and images were captured with ChemiDocTM XRS+ (Bio-Rad, USA).

### Safety evaluation in vitro and vivo

A hemolysis assay was conducted by acquiring pure RBCs from SD rats. Subsequently, a 5% RBC suspension (volume/volume, v/v) was prepared by mixing SIN, GP NPs, or HA@RFM@GP@SIN NPs (12.5, 25, 50, 100, and 200 µg/mL GP) with PBS. Mixture incubation was implemented at 37 °C for 4 h, followed by centrifugation at 3000 rpm for 5 min. The supernatants were collected and analyzed using a microplate detector (Epire, USA) at 540 nm. The hemolysis rate was calculated utilizing Eq. (3):

3 $${\text{Hemolysis}}\,{\text{rate}}\,{\text{(\%)}}\,{\text{ = }}\frac{{\text{h}}}{{{\text{h0}}}}\,{ \times100\% }$$

where h denotes the absorbance value of supernatant in a drug group, and h_0_ signifies the absorbance value of RBC in water.

RBC forms were observed with the help of a phase contrast microscope (Olympus, Tokyo, Japan).

A coagulation assay was performed by collecting platelet-rich plasma from SD rats using whole blood. Next, 200 µg/mL of SIN, GP NPs, or HA@RFM@GP@SIN NPs were added to the platelet-rich plasma and incubated at 37 °C for 1 h. The absorbance at 650 nm was tested, and the coagulation rate was calculated.

Cell cytotoxicity assay was conducted using H9C2, HL7702, HK2, VSMC, HUVEC, RAW264.7, and HFLS cells. The cells were incubated in 10% FBS for 24 h, then treated with 1% FBS containing GP NPs or HA@RFM@GP@SIN NPs (20, 40, 80, and 160 µg/mL GP) for further 24 h. Cell viability was tested employing the MTT assay and calculated with the help of Eq. (4).

4 $${\text{Cell}}\,{\text{viability}}\,{\text{ = }}\frac{{{\text{OD490}}\,{\text{nm(sample)}}}}{{{\text{OD490}}\,{\text{nm(control)}}}}\,{ \times100\%}$$

Evaluation of immune escape was performed by culturing RAW264.7 cells (1.5 × 10^5^ cells/well) in 10% FBS for 24 h. Subsequently, Rho-labeled GP NPs and HA@RFM@GP NPs (40, 80, 160 µg/mL GP) were added to the plate and incubated for 4 h. The resulting images were observed using CLSM.

Animal safety and toxicity testing involved sacrificing the animals on day 36 after the CFA injection. Blood samples were harvested from the abdominal aorta of the AIA rats. Among these samples, whole blood was selected for blood routine analysis, while plasma was subjected to liver and kidney function detection using a biochemical analyzer. The heart, liver, spleen, lungs, and kidneys were fixed with 4% paraformaldehyde, paraffin-embedded, sectioned, and stained with H&E.

### Statistical analysis

Results were reported as the average ± standard error of the mean (SEM) based on individual experiments. For the statistical evaluations, comparisons between two sets were implemented utilizing Student’s two-sided t-test or one-way analysis of variance (ANOVA) as applicable, followed by a post hoc examination applying the least significant difference (LSD) method for multiple group analyses. *P* < 0.05 was deemed as statistical significance. All the statistical procedures were executed utilizing GraphPad Prism version 8.0 software (La Jolla, CA, USA).

## Results and discussion

### Fabrication and characterization of RFM and HA@RFM@GP@SIN NPs

**Fig. 1:**
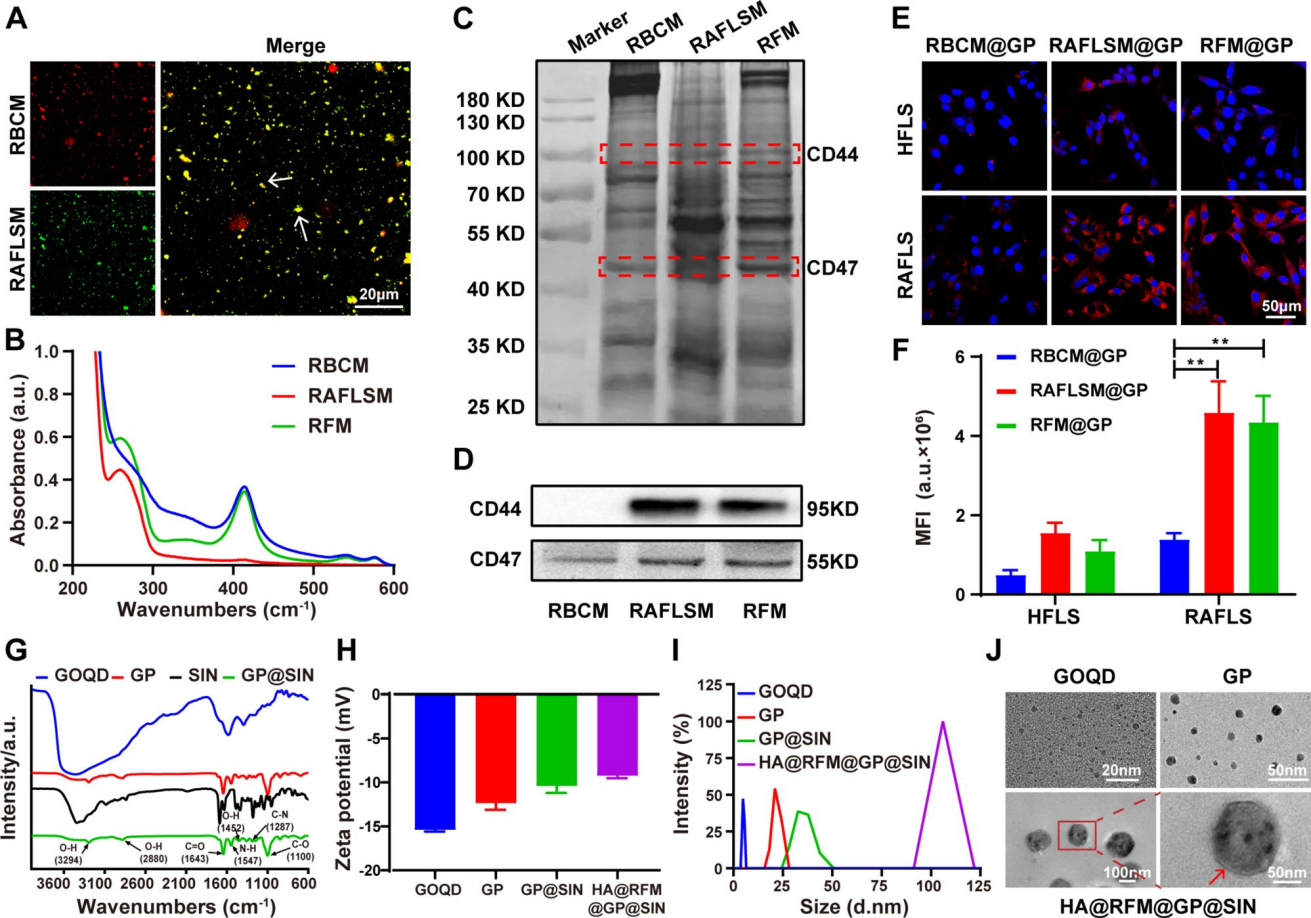
Fabrication and characterization of RFM and HA@RFM@GP@SIN NPs. (**A**) Confocal fluorescence microscopic images of the RFM (Green = RAFLSM, Red = RBCM; scale bar = 20 μm). (**B**) The UV-*vis* spectra of RBCM, RAFLSM, and RFM. (**C**) SDS-PAGE protein analysis of membrane protein. (**D**) WB analysis of characteristic RAFLSM marker CD44 and RBCM marker CD47. (**E**) The representative images and (**F**) quantitative data of RBCM@GP NPs, RAFLSM@GP NPs, and RFM@GP NPs uptake by HFLS and RAFLS, respectively. Scale bar = 50 μm. Data are the means ± SEM, *n* = 3 per treatment, ^**^P < 0.01. (**G**) Fourier transform infrared spectroscopy of GOQD, GP NPs, SIN, and GP@SIN NPs. (**H**) The surface zeta potentials and (**I**) size distribution of GOQD, GP NPs, GP@SIN NPs, HA@RFM@GP@SIN NPs. (**J**) TEM images of GOQD, GP NPs, and HA@RFM@GP@SIN NPs

At first, we prepared hybrid membrane for NPs coating. A membrane fusion assay was conducted to investigate the hybridization efficiency of DiI-labeled RBCM (red) and DiO-labeled RAFLSM membranes (green). As depicted in Fig. 1A, the colocalization of green and red dots resulted in the formation of yellow dots, which was frequently observed for RFM. The UV-vis spectra presented in Fig. 1B confirmed the characteristic peaks of RAFLSM and RBCM within RFM, located at wavelengths 260 nm and 412 nm, respectively. Subsequently, RFM properties were thoroughly characterized. SDS-PAGE analysis in Fig. 1C demonstrated that RFM retained all membrane proteins from RBCM and RAFLSM. Furthermore, WB analysis revealed the presence of CD44 (a RAFLSM marker) and CD47 (an RBC marker) in RFM, while RBCM only expressed CD47 and RAFLSM predominantly expressed CD44 (Fig. 1D). Importantly, the uptake efficiency of RFM-coated GP NPs was investigated by HFLS and RAFLS. As shown in Fig. 1E and F, the RAFLSM@GP NP and RFM@GP NP groups exhibited strong red fluorescence in RAFLS, while minimal fluorescence was observed in RBCM@GP NP-treated RAFLS. Additionally, weak fluorescence was witnessed in the HFLS among these three groups, highlighting the preferential internalization of RFM@GP NPs by RAFLS. Collectively, these findings confirm the successful hybridization of RBCM and RAFLSM to produce RFM, which possesses the characteristics of both RBCM and RAFLSM.

Subsequently, the loading and encapsulation efficacy of SIN in GP NPs were investigated. The results indicated that a mass ratio of GP to SIN of 1:3 achieved optimal load capacity (103.10%) and encapsulation capacity (28.96%), indicating that approximately 1 mg of GP could be loaded with 1 mg of SIN (Fig. S1). The FT-IR spectrum of GP@SIN NPs exhibited characteristic peaks of GP (1547, 1452, and 1100 cm^− 1^) and SIN (3294, 2880, 1643, and 1287 cm^− 1^), confirming the successful loading of SIN in the GP NPs (Fig. 1G). Surface zeta potential analysis revealed that GOQD, GP NPs, GP@SIN NPs, and HA@RFM@GP@SIN NPs had zeta potentials of -15.40 ± 0.14 mV, -12.37 ± 0.61 mV, -10.38 ± 1.38 mV, and − 9.23 ± 0.23 mV, respectively (Fig. 1H). The increased potential from GP NPs to HA@RFM@GP@SIN NPs can be attributed to the positive charges of SIN [14] and DSPE-PEG_2000_-NH_2_ [25]. Particle size analysis demonstrated that the average diameters of GOQD, GP NPs, GP@SIN NPs, and HA@RFM@GP@SIN NPs were 4.36 ± 0.46 nm (Polydispersity index (PDI): 0.44 ± 0.10), 21.19 ± 1.14 nm (PDI: 0.36 ± 0.03), 37.04 ± 1.48 nm (PDI: 0.47 ± 0.05), and 115.00 ± 3.86 nm (PDI: 0.37 ± 0.05), respectively (Fig. 1I). TEM images in Fig. 1J revealed that the HA@RFM coatings encapsulated numerous GP NPs, resulting in an approximately 5.5-fold increase in particle size from GP NPs to HA@RFM@GP@SIN NPs, consistent with the above size assay.

### Cellular uptake of HA@RFM@GP@SIN NPs in vitro

**Fig. 2:**
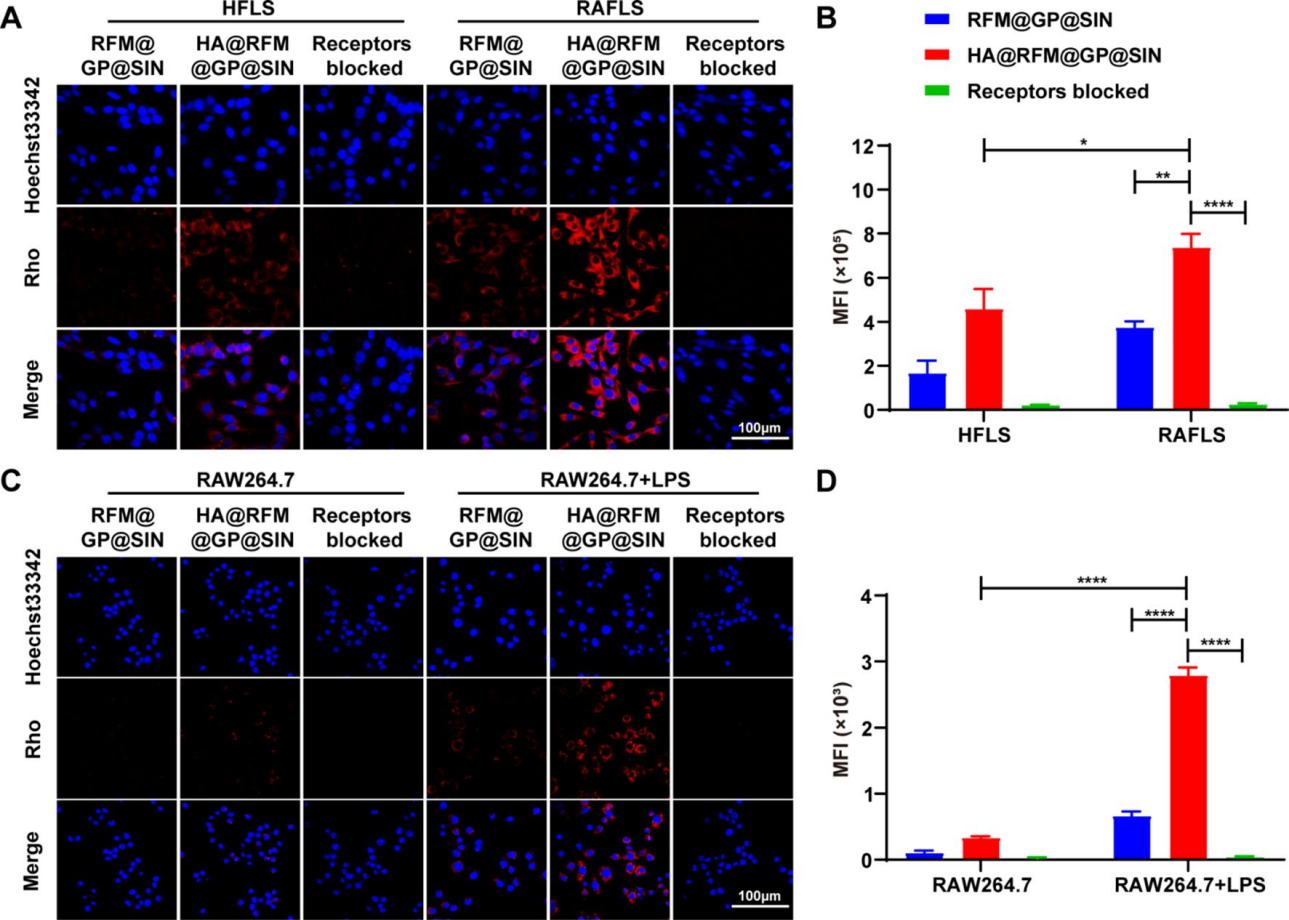
Cellular uptake of HA@RFM@GP@SIN NPs in vitro. (**A**) Representative images of CLSM and (**B**) quantification analysis of HFLS or RAFLS incubated with RFM@GP@SIN NPs and HA@RFM@GP@SIN NPs. (**C**) Representative images of CLSM and (**D**) quantification analysis of RAW264.7 or LPS-activated RAW264.7 incubated with RFM@GP@SIN NPs and HA@RFM@GP@SIN NPs. Data are the means ± SEM, *n* = 3 per treatment, ^*^P < 0.05, ^**^P < 0.01, ^****^P < 0.0001

The efficacy of nanodrug delivery systems depends on their ability to efficiently enter cells [26]. To assess the cellular uptake efficiency of HA@RFM@GP@SIN NPs, HFLS, RAFLS, RAW264.7, and LPS-activated RAW264.7 cells were employed. The findings indicated that a significant number of HA@RFM@GP@SIN NPs were absorbed by RAFLS, as evidenced by the intense red fluorescence signals. Conversely, HFLS exhibited limited uptake of HA@RFM@GP@SIN NPs, resulting in weak fluorescence (Fig. 2A and B). Furthermore, when RAFLS and HFLS were co-incubated with RFM@GP@SIN NPs or a receptor-blocker, negligible fluorescence was observed. Notably, macrophages also displayed red fluorescence signals, following a similar trend as RAFLS (Fig. 2C and D). These outcomes indicate that RAFLS and activated macrophages exhibit high efficiency in the uptake of HA@RFM@GP@SIN NPs.

Understanding the mechanisms of uptake and localization of NPs within cells is crucial for their safe and efficient application [27]. Hence, in this study, we employed small-molecule inhibitors to delineate the uptake mechanism of HA@RFM@GP@SIN NPs into RAFLS. As depicted in Fig. S2A and B, the presence of inhibitors such as M-β-CD, and colch, resulted in a reduction of HA@RFM@GP@SIN NP uptake to 70.93 ± 6.16% and 46.58 ± 3.70%, respectively. This finding is consistent with a prior report that membrane-coated GP is mainly internalized through caveolae endocytosis [28]. In the absence of inhibitors, HA@RFM@GP@SIN NPs (labeled with Rho) were found to localize in the lysosomes after 2, 4, 6, and 8 h of exposure (Fig. S3). At 2 h, colocalization between HA@RFM@GP@SIN NPs and lysosomes occurred and reached the plateau at 6 h (indicated by yellow fluorescence). Subsequently, at 8 h, partial red fluorescence appeared in the cytoplasm, suggesting the successful escape of HA@RFM@GP@SIN NPs from the lysosomes at 6 h. This is of significant importance in reducing drug degradation within the acidic lysosomal environment and thus enhancing the therapeutic efficacy [23].

### M1 to M2 reporlariztion of macrophages caused by HA@RFM@GP@SIN NPs in vitro

**Fig. 3:**
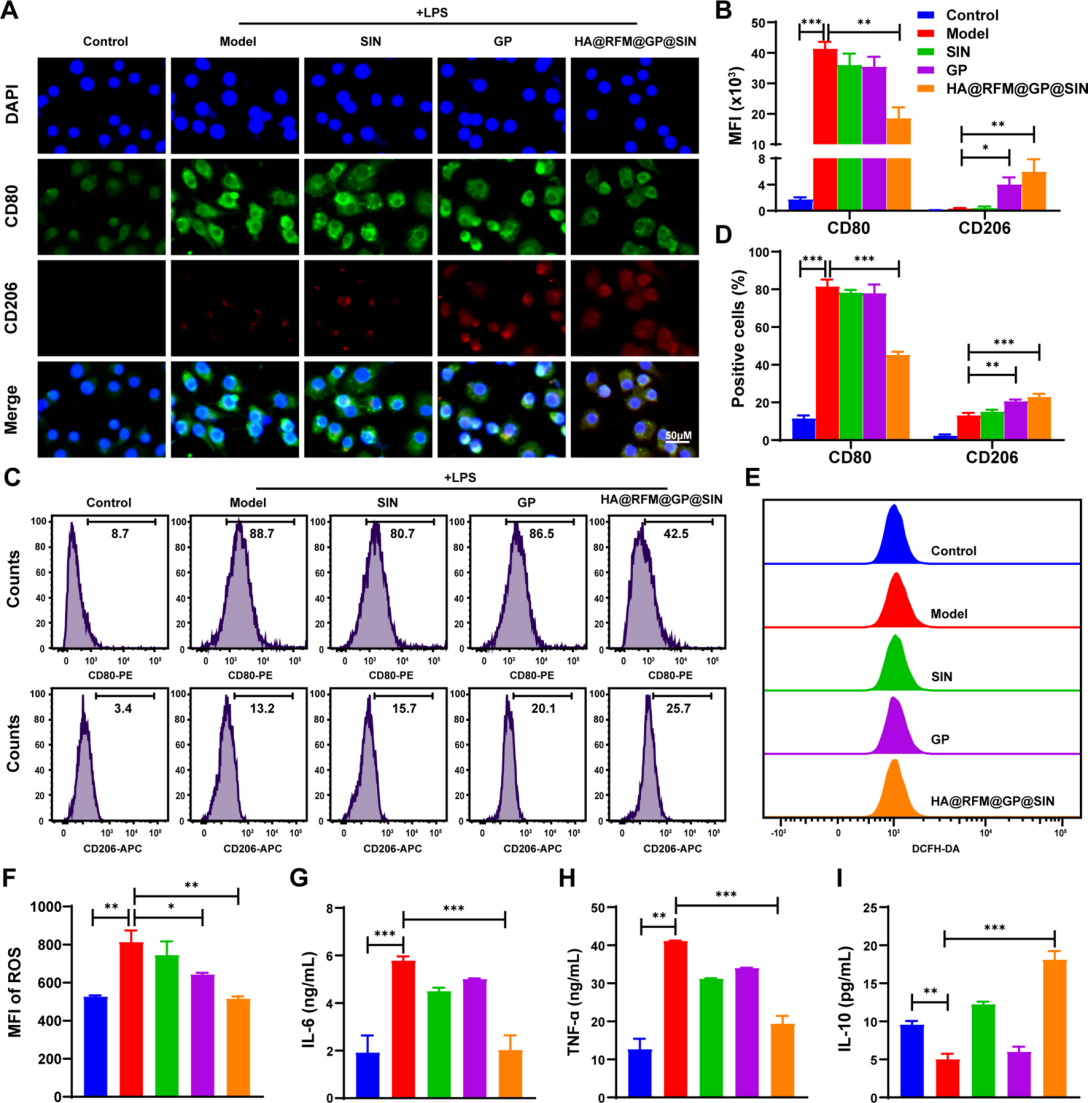
M1 to M2 reporlariztion of macrophages caused by HA@RFM@GP@SIN NPs in vitro. (**A**) Immunofluorescence staining and (**B**) MFI values of CD80 (green), CD206 (red), and nuclei (blue) on macrophages incubated with PBS, SIN, GP NPs, or HA@RFM@GP@SIN NPs after 48 h. (**C**) Percentages of CD80- or CD206-positive macrophages were analyzed by flow cytometry and (**D**) quantified. (**E**) ROS detection and (**F**) quantification in macrophages were performed by flow cytometry. Quantitative analysis of (**G**) IL-6, (**H**) TNF-α, and (**I**) IL-10 levels in macrophages by ELISA. Data are the means ± SEM, *n* = 3 per treatment, ^*^P < 0.05, ^**^P < 0.01, ^***^P < 0.001

Macrophages, being the most abundant immune cells in inflamed joint, exhibit two distinct phenotypes: M1 macrophages, which serve as the primary source of pro-inflammatory cytokines, and M2 macrophages, which secrete anti-inflammatory cytokines to alleviate inflammation [29]. Therefore, the strategic modulation of macrophage polarization from M1 to M2 phenotype represents an efficient approach for treating RA [30, 31]. To probe into the regulatory effect of HA@RFM@GP@SIN NPs on macrophage polarization, the expression of macrophage surface markers, CD80 (M1 phenotype) and CD206 (M2 phenotype) was analyzed via immunofluorescence staining and flow cytometry. As shown in Fig. 3A, when macrophages were polarized towards the M1 phenotype using LPS, robust upregulation in the green fluorescence intensity of CD80 was observed. Conversely, following treatment with HA@RFM@GP@SIN NPs, the CD80 signal decreased while the expression of CD206 (red fluorescence) became more prominent, indicating the effective repolarization of macrophages from M1 to M2 phenotype (Fig. 3B). Flow cytometry analysis obtained consistent results (Fig. 3C and D). As anticipated, upon LPS-induced polarization, the proportion of M1 macrophages significantly increased to 88.7% relative to the control. However, upon intervention with HA@RFM@GP@SIN NPs, the proportion of M1 macrophages dramatically decreased to 42.5% within 48 h, while the proportion of M2 macrophages rose from 3.4 to 25.7%.

Furthermore, the underlying mechanism of HA@RFM@GP@SIN NPs on anti-inflammatory is still under research. DCFH-DA probe was employed to detect the effect of different NPs on reactive oxygen species (ROS) levels in inflammatory macrophages. As we expected, activated macrophages showed high-level ROS, which was reflected by the strong fluorescence of cells emitted from of DCFH-DA. Strikingly, the phenomenon was reversed by treatment with GP NPs or HA@RFM@GP@SIN NPs. Notably, the ROS scavenging effect of HA@RFM@GP@SIN NPs was greater than GP NPs (Fig. 3E and F). Additionally, the anti-inflammatory characterization of HA@RFM@GP@SIN NPs in macrophages, including IL-6, TNF-α (M1 markers), and IL-10 (M2 marker), was quantified by ELISA. The results showed that HA@RFM@GP@SIN NPs notably reduced the levels of IL-6 (Fig. 3G) and TNF-α (Fig. 3H) while enhancing the expression of IL-10 (Fig. 3I). These findings suggest that NPs can modulate the transition of macrophages from the M1 to the M2 phenotype, thereby exerting antioxidant and anti-inflammatory effects.

### HA@RFM@GP@SIN NPs inhibited RAFLS proliferation in vitro

**Fig. 4:**
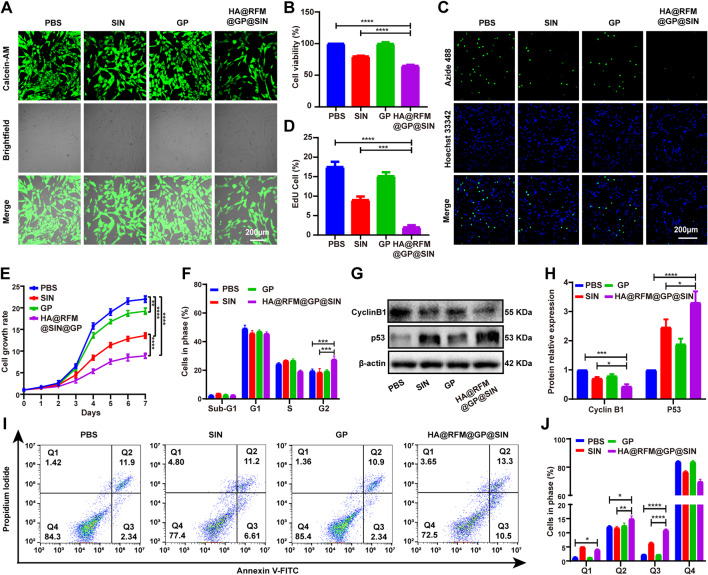
HA@RFM@GP@SIN NPs inhibited RAFLS proliferation in vitro. (**A**) Fluorescence imaging of live RAFLS cells incubated with PBS, SIN, GP NPs, or HA@RFM@GP@SIN NPs after 48 h. (**B**) MTT proliferation assay of RAFLS incubated with various formulations after 48 h. (**C**) Fluorescence image and (**D**) quantitative analysis of EdU proliferation assay of RAFLS treated with PBS, SIN, GP NPs, and HA@RFM@GP@SIN NPs. (**E**) Cell growth curves of RAFLS in different treatment groups. (**F**) Cell cycle distribution of RAFLS after incubation with various formulations. (**G**) WB and (**H**) quantitative analyses of cyclin B1 or p53 in RAFLS after treatment with PBS, SIN, GP NPs, and HA@RFM@GP@SIN NPs. (**I**) Flow cytometry and (**J**) quantitative analyses of apoptosis in RAFLS. Data are the means ± SEM, *n* = 3 per treatment, ^*^P < 0.05, ^**^P < 0.01, ^***^P < 0.001, ^****^P < 0.0001

Then, the viability of RAFLS treated with HA@RFM@GP@SIN NPs was assessed to investigate the impact of these NPs on the cell proliferation. As anticipated, the number of viable cells in the HA@RFM@GP@SIN group significantly decreased, as observed through staining of viable cells (Fig. 4A). The MTT assay further confirmed that HA@RFM@GP@SIN NPs treatment considerably reduced the viability of RAFLS, compared to the PBS group. Notably, HA@RFM@GP@SIN NPs (containing 100 µM SIN) at the same dose exhibited a stronger suppressive effect on RAFLS viability than SIN alone (100 µM) (Fig. 4B). Additionally, the EdU proliferation assay was employed to assess individual cell proliferation. Both the fluorescence image (Fig. 4C) and the quantitative analysis (Fig. 4D) revealed a significant decline in the number of proliferating cells after with HA@RFM@GP@SIN NPs treatment. Furthermore, the cell growth curve illustrated an initial lag phase of 0–2 days, a logarithmic phase of 2–6 days, and a stationary phase of 6–7 days for RAFLS growth. Notably, during the logarithmic growth period of 2–6 days, the growth rates gradually declined in the PBS, SIN, and HA@RFM@GP@SIN groups, respectively (Fig. 4E). In comparison to day 0, the growth rate on day 7 was 22.00 ± 0.27 times for the PBS group, whereas for the SIN and HA@RFM@GP@SIN groups, it was only 13.60 ± 0.18 times and 8.91 ± 0.41 times, respectively. Subsequently, potential reasons for the inhibition of RAFLS proliferation in the HA@RFM@GP@SIN group were evaluated. Flow cytometry assays were employed to explain the cell cycle stage of RAFLS (Fig. S4), and the cell cycle of the HA@RFM@GP@SIN-treated group was arrested into G2 phase (Fig. 4F). WB was conducted to measure the protein expression of G2 phase markers, cyclin B1 and p53. The HA@RFM@GP@SIN group exhibited increased levels of p53 protein expression and decreased levels of cyclin B1 protein expression compared to the PBS and SIN groups (Fig. 4G and H). Moreover, flow cytometry analysis demonstrated that HA@RFM@GP@SIN NPs also heightened the proportion of RAFLS in early (Q3) and late (Q2) apoptosis (Fig. 4I and J). These experiments conclusively demonstrated that HA@RFM@GP@SIN NPs hindered proliferation in the G2 phase of the cell cycle and promoted apoptosis of RAFLS.

### Pharmacokinetics and biodistribution of HA@RFM@GP@SIN NPs in AIA rats

**Fig. 5:**
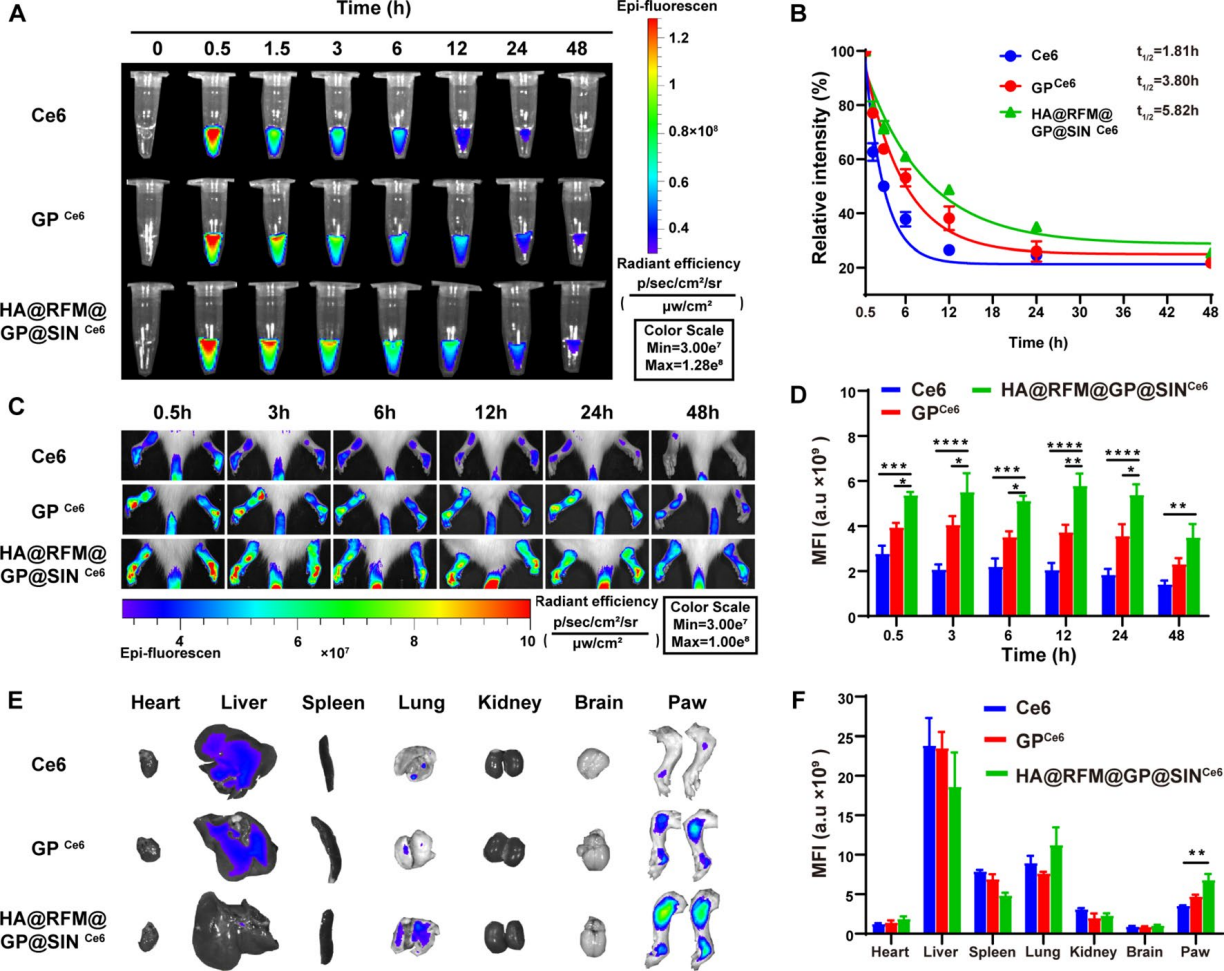
Pharmacokinetics and biodistribution of HA@RFM@GP@SIN NPs in AIA rats. (**A**) The fluorescence images and (**B**) pharmacokinetic curves of plasma samples collected from AIA rats after intravenous administration of Ce6, GP^Ce6^ NPs, and HA@RFM@GP@SIN^Ce6^ NPs at different time points. (**C**) Fluorescent distribution and (**D**) quantitation of NPs at various time points in the arthritis site. (**E**) Fluorescent distribution and (**F**) quantification of NPs in major organs at 48 h post-injection. Data are the means ± SEM, *n* = 3 per treatment, ^*^P < 0.05, ^**^P < 0.01, ^***^P < 0.001, ^****^P < 0.0001

The pharmacokinetics of HA@RFM@GP@SIN NPs in rats with AIA were investigated using the Ce6 fluorescence labeling method [32]. Compared to GP^Ce6^ NPs (5.82 h vs. 3.80 h) and free Ce6 (5.82 h vs. 1.81 h), the circulation half-life (t_1/2_) of HA@RFM@GP@SIN^Ce6^ NPs in AIA rats increased by 1.53- and 3.22-fold, respectively (Fig. 5A and B). This result demonstrates the prolonged half-life of GP NPs after being coated with RFM. Based on these promising results, the targeting ability of GP^Ce6^ NPs or HA@RFM@GP@SIN^Ce6^ NPs for arthritic hind paws was evaluated precisely. Only weak fluorescence was detected in arthritic paws due to the rapid metabolism and non-specific targeting of GP^Ce6^ NPs. However, HA@RFM@GP@SIN^Ce6^ NPs administration resulted in strong fluorescent signals emitted from the arthritic paws (Fig. 5C). An increase in fluorescence intensity was seen in arthritic paws treated with HA@RFM@GP@SIN^Ce6^ NPs compared to those treated with GP^Ce6^ NPs at different time points (Fig. 5D). These results indicate the highly specific accumulation of HA@RFM@GP@SIN^Ce6^ NPs at the arthritic sites. Additionally, ex vivo fluorescence images showed partial accumulation of HA@RFM@GP@SIN^Ce6^ NPs in the lung (Fig. 5E and F), which aligns with a previous report that HA@RFM@GP@SIN^Ce6^ NPs with a small particle size can be taken up by the endothelial cells of the lung [33].

### HA@RFM@GP@SIN NPs inhibited synovial hyperplasia, facilitated the transition of M1 to M2 macrophages, and prevented cartilage erosion and bone destruction in AIA rats

**Fig. 6:**
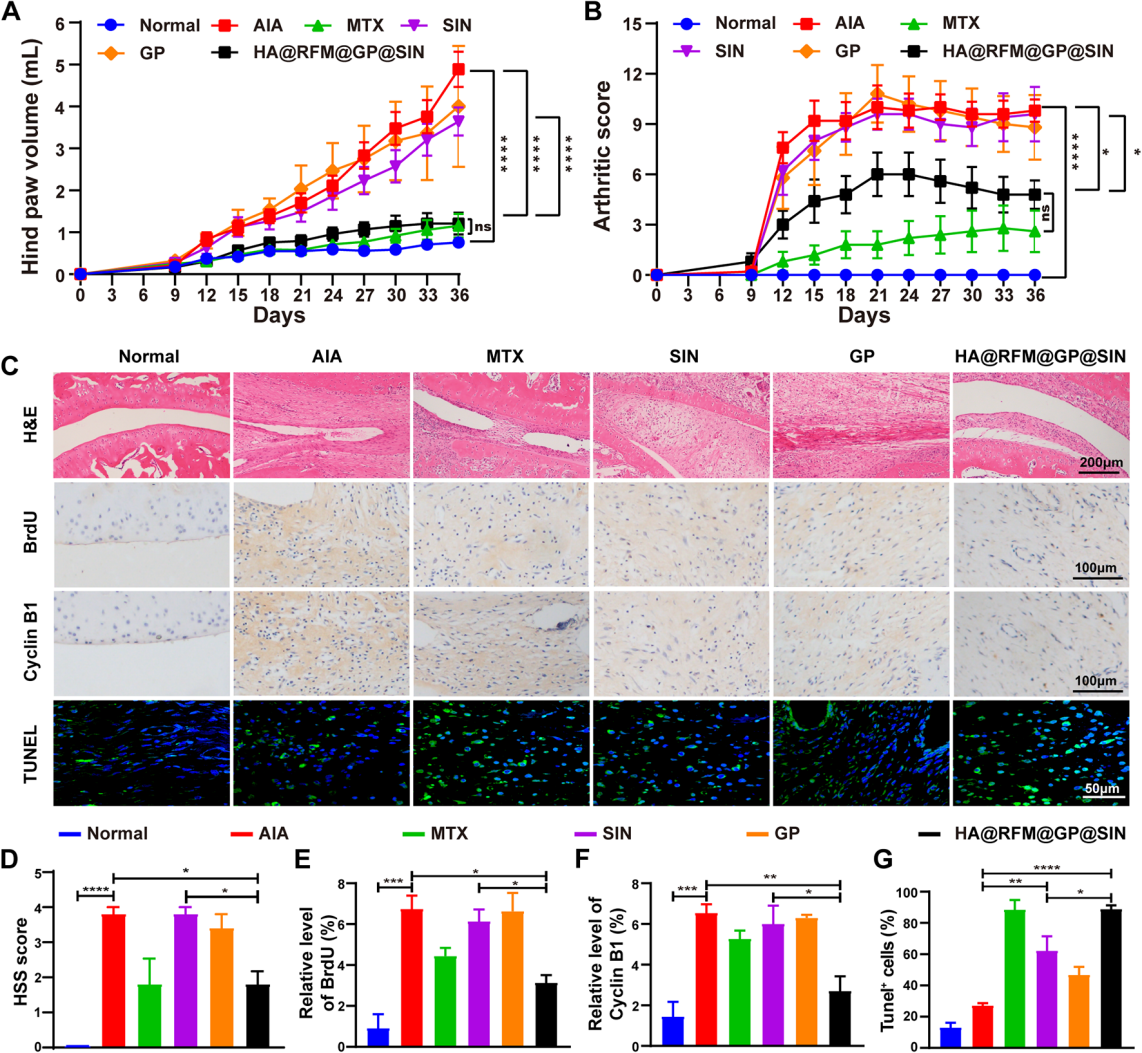
HA@RFM@GP@SIN NPs inhibited synovial hyperplasia in AIA rats. (**A**) Hind paw swelling volume and (**B**) arthritis scores in AIA rats treated with PBS, MTX, SIN, GP NPs, or HA@RFM@GP@SIN NPs. (**C**) Representative images of the H&E, BrdU, cyclin B1, and TUNEL staining. (**D**) The quantitative analysis of HSS, (**E**) BrdU, (**F**) cyclin B1, and (**G**) TUNEL in ankle synovium. Data are presented as the means ± SEM (*n* = 5 per formulation), ns stands for no significance, ^*^P < 0.05, ^**^P < 0.01, ^***^P < 0.001, ^****^P < 0.0001

Then AIA rat models were utilized to assess the therapeutic potential of HA@RFM@GP@SIN NPs [22]. MTX was used as a positive control to validate the success of the AIA model in our study, it is the most commonly used disease-modifying antirheumatic drug for therapy of RA [34]. However, long-term use of MTX is impeded by its frequent adverse reactions and potential toxic effects (e.g., hepatotoxicity, nephrotoxicity, or even malignant diseases) [35, 36]. As depicted in Fig. 6A and B, the tail vein administration of SIN at 6 mg/kg alone failed to significantly alleviate arthritic symptoms in the AIA rats. However, after loading SIN into the developed NPs, the caudal intravenous administration of HA@RFM@GP@SIN NPs (containing 6 mg/kg SIN) every three days from days 0 to 36 significantly reduced hind paw volume and decreased the arthritic score, which is comparable to that of MTX. This observation was further supported by H&E staining, immunohistochemical analysis, and TUNEL staining, which confirmed that HA@RFM@GP@SIN NPs inhibited the abnormal proliferation of RAFLS (Fig. 6C). H&E staining illustrated severe synovial hyperplasia in the joint cavity of the AIA group, with the synovium completely occluding the joint cavity. In contrast, this phenomenon was ameliorated in the HA@RFM@GP@SIN group (Fig. 6D). Immunohistochemical detection of BrdU in ankle synovial tissue, as a marker of individual cell proliferation, revealed reduced BrdU expression in the HA@RFM@GP@SIN group compared to the AIA and SIN groups (Fig. 6E). Additionally, the inhibition of cyclin B1 expression was found in the synovium of the HA@RFM@GP@SIN group (Fig. 6F). TUNEL staining indicated that HA@RFM@GP@SIN induced synovial cell apoptosis, the rate of which is higher than that of SIN (Fig. 6G). Thus, the findings indicated a significant reduction in proliferating synovial cells in the HA@RFM@GP@SIN group compared to both the AIA and SIN groups.

**Fig. 7:**
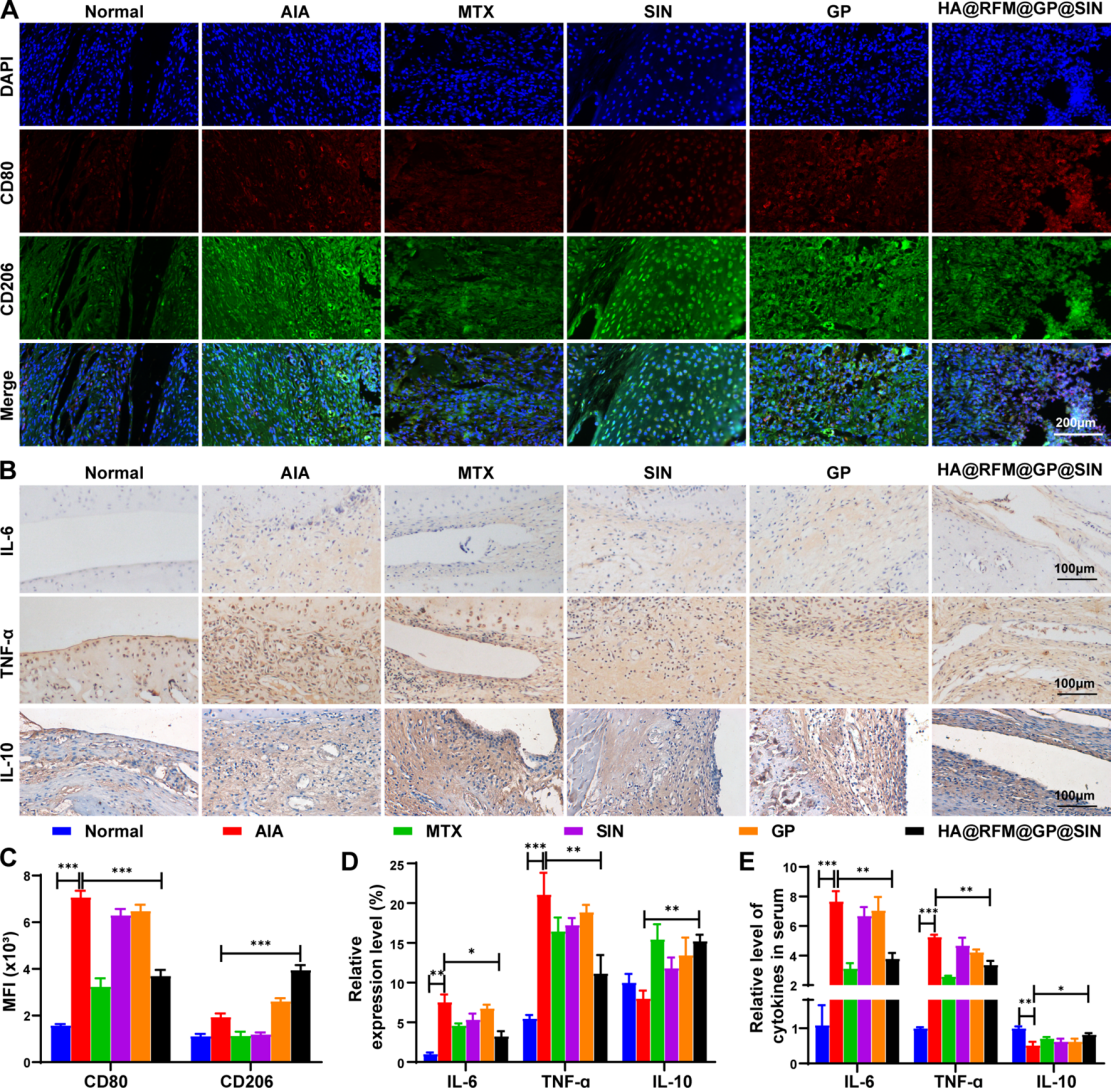
HA@RFM@GP@SIN NPs facilitated the transition of M1 to M2 macrophages in AIA rats. (**A**) Immunofluorescence and (**C**) quantitative analysis of M1 (CD80, green) and M2 (CD206, red) macrophage markers in the joint after treatment. (**B**) Immunohistochemical images and (**D**) quantitative analysis of IL-6, TNF-α, and IL-10 in ankle synovium. (**E**) Quantitative analysis of IL-6, TNF-α, and IL-10 levels in serum of AIA rats after various treatments. Data are presented as the means ± SEM (*n* = 3 per formulation), ^*^P < 0.05, ^**^P < 0.01, ^***^P < 0.001

In RA joints, macrophages predominantly exhibit the M1 phenotype, which contributes to the progression of RA through the secretion of various inflammatory cytokines [37]. As a result, we evaluated the phenotypic transition of synovial macrophages by conducting immunofluorescent staining for M1 (CD80) and M2 (CD206) markers. The level of CD80 (green) signal in inflamed joints significantly increased compared to that in the normal group. However, intravenous administration of HA@RFM@GP@SIN NPs effectively reduced the level of CD80 while simultaneously increasing the presence of CD206 (red), indicating successful repolarization of macrophages from the M1 to M2 phenotype (Fig. 7A and C). Accordingly, immunohistochemistry in Fig. 7B demonstrated that the synovium levels of IL-6 and TNF-α were notably higher in the AIA group relative to the normal group. However, treatment with HA@RFM@GP@SIN NPs reduced the expression of these markers (Fig. 7D). Notably, the synovium levels of IL-10 increased in the HA@RFM@GP@SIN group. Additionally, ELISA assay (Fig. 7E) highlighted the same trend as the observed therapeutic efficacy, further confirming that HA@RFM@GP@SIN NPs exert anti-inflammatory activity by modulating macrophage polarization and subsequently regulating the expression of inflammatory cytokines.

**Fig. 8:**
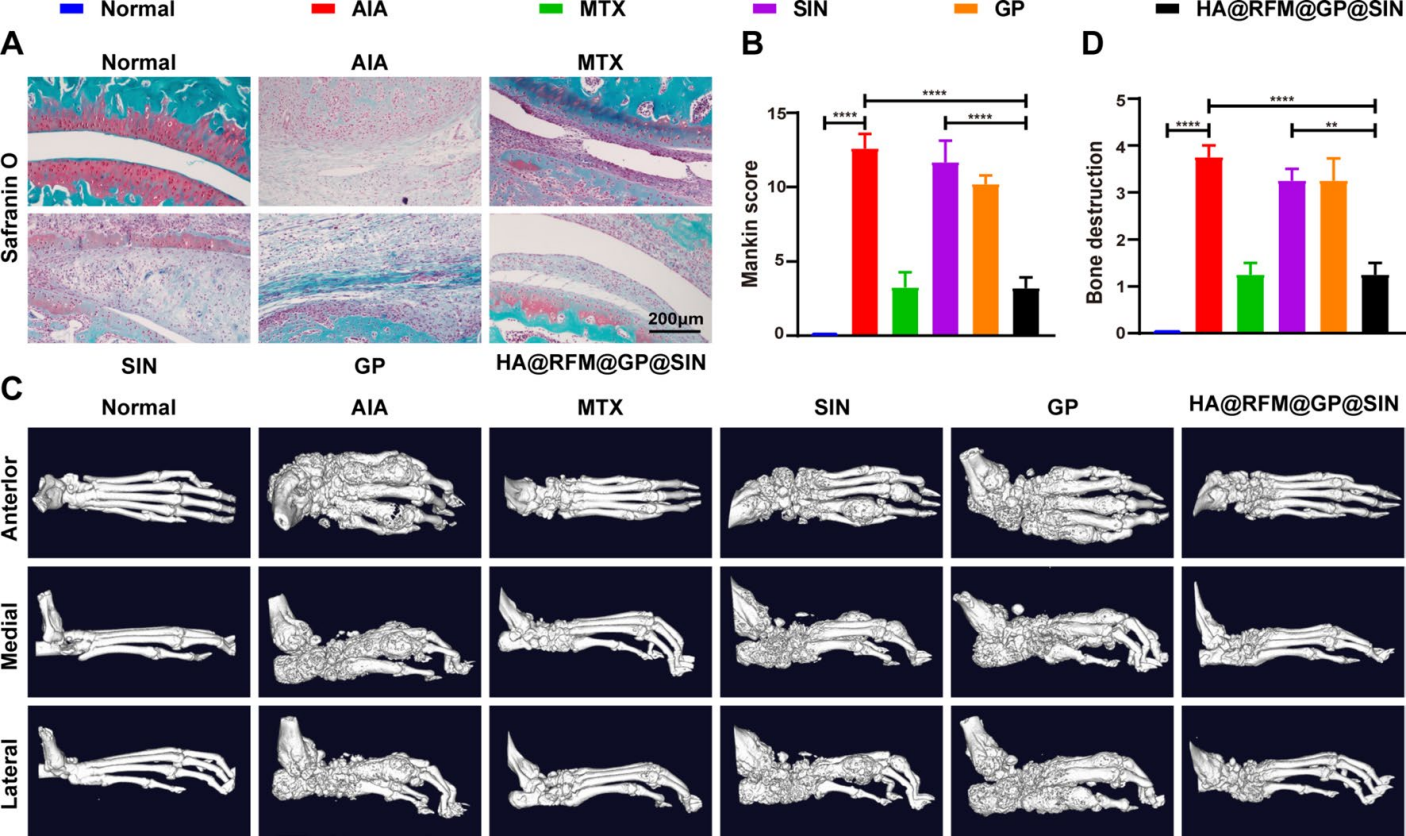
HA@RFM@GP@SIN NPs prevented cartilage erosion and bone destruction in AIA rats. (**A**) Representative images of the Safranin O staining and (**B**) the Mankin score of paws of AIA rats with treatment. (**C**) Micro-CT radiology score of paws and (**D**) bone destruction scores in AIA with various treatments. Data are presented as the means ± SEM (*n* = 3 per formulation), ^*^P < 0.05, ^**^P < 0.01, ^***^P < 0.001, ^****^P < 0.0001

The destruction of bone and cartilage in RA joints is primarily driven by synovial fibroblasts and macrophages, which can influence osteoclastogenesis by releasing matrix-degrading enzymes and pro-inflammatory factors [38]. By utilizing safranin O staining and micro-CT imaging to illustrate the efficacy of HA@RFM@GP@SIN NPs on cartilage and bone damage in inflamed ankle joints, we found revealed significant destruction of the cartilage structure, notable reduction in chondrocyte count, and loss of the cartilage matrix in the AIA group (Fig. 8A). However, AIA rats treated with HA@RFM@GP@SIN NPs displayed marginally lower severity of these symptoms, resulting in reduced synovial inflammation and cartilage loss (Fig. 8B). Micro-CT images (Fig. 8C) and bone destruction scores (Fig. 8D) further substantiated the findings, which was reflected by rough joint surfaces, prominent marginal osteophytes, narrowed joint cavities, and impaired interphalangeal and metatarsal joint correspondence in the AIA group. These observations exhibited greater improvement in the HA@RFM@GP@SIN group compared to both the SIN and GP groups. In summary, these results ascertained the effectiveness of HA@RFM@GP@SIN NPs in preventing cartilage erosion and bone destruction in the AIA rat model.

### Anti-arthritic effects and metabolomics analysis of HA@RFM@GP@SIN NPs in CIA rats

**Fig. 9:**
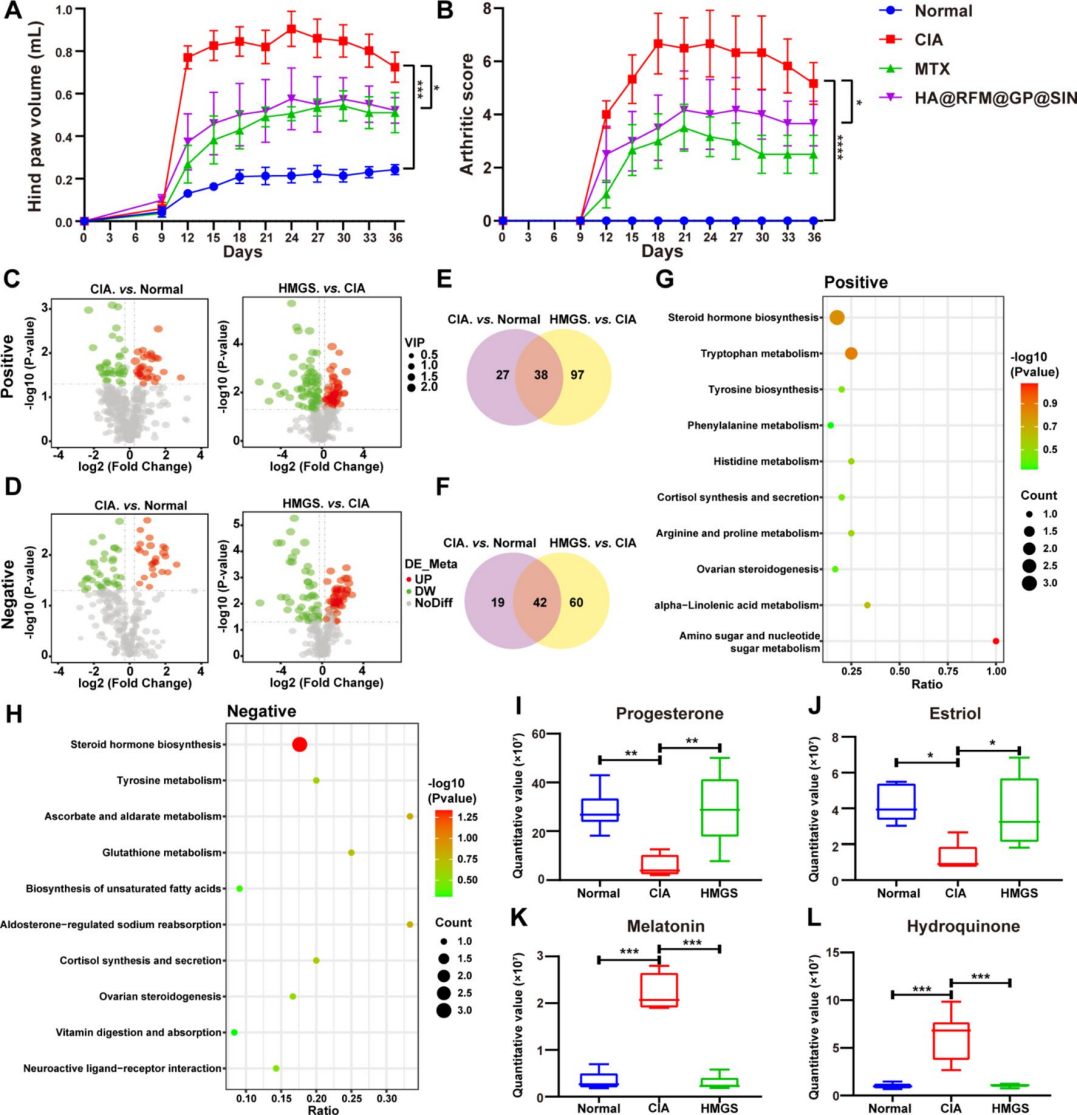
Anti-arthritic effects and metabolomic analysis of HA@RFM@GP@SIN NPs in CIA rats. (**A**) Hind paw swelling volume and (**B**) arthritis scores in CIA rats treated with PBS, MTX, or HA@RFM@GP@SIN NPs. (**C**) Volcano plot analysis of metabolites between the CIA and normal groups, as well as between the CIA and HA@RFM@GP@SIN NPs (HMGS) groups under positive and (**D**) negative ion modes. (**E**) Venn diagram of differential metabolites among normal, CIA, and HMGS groups under positive and (**F**) negative ion modes. (**G**) KEGG pathway analysis of differential metabolites among normal, CIA, and HMGS groups under positive and (**H**) negative ion modes. (**I**) Expression of progesterone, (**J**) estriol, (**K**) melatonin, and (**L**) hydroquinone in different groups. Data are presented as the means ± SEM (*n* = 6 per formulation), ^*^P < 0.05, ^**^P < 0.01, ^***^P < 0.001

The pathogenesis of RA is complex and not fully understood [39]. Similar to many other autoimmune diseases, RA affects women two to three times more frequently than men, with the risk increasing with age [40]. Therefore, employing suitable RA models is essential for understanding the disease’s pathology, investigating drug actions, and advancing new treatments. CIA models, reflecting similar immunological and pathological features as human RA, are widely used for studying autoimmune mechanisms [41]. To bridge the gap between laboratory findings and clinical applications, female rats were selected as research subjects in this study to establish a CIA model, thus enabling the examination of the potential therapeutic mechanisms of HA@RFM@GP@SIN NPs. As depicted in Fig. 9A and B, paw volumes increased three-fold, and the arthritic score exhibited a difference of more than five points in the CIA group relative to the normal group. In contrast, both paw swelling and the arthritic score were declined in the group treated with HA@RFM@GP@SIN NPs, demonstrating a similar trend to the results obtained in the AIA model and further confirming the therapeutic efficacy of HA@RFM@GP@SIN NPs.

Metabolomic analysis was implemented to test the effect of HA@RFM@GP@SIN NPs on RA, specifically focusing on serum metabolites. Plasma samples were collected from three groups: normal (normal group), CIA, and HA@RFM@GP@SIN-treated (HMGS group) CIA rats. Partial least squares discriminant analysis (PLS-DA) was performed for all three groups, revealing significant differences between them in both positive and negative ion modes (Fig. S5A and 5B). The volcano diagram displayed the distribution of differential metabolites between the CIA and normal groups, as well as between the HA@RFM@GP@SIN and CIA groups, under both ion modes (Fig. 9C and D). In the diagram, upregulated metabolites were denoted in red, while downregulated metabolites were shown in green. The positive ion mode analysis revealed 38 shared differential metabolites among the three groups (Fig. 9E), while the negative ion mode analysis indicated 42 common differential metabolites (Fig. 9F). Additionally, Kyoto Encyclopedia of Genes and Genomes (KEGG) pathway enrichment analysis, based on the shared differential metabolism, demonstrated the involvement of steroid hormone biosynthesis, tyrosine metabolism, and ovarian steroidogenesis in RA treated with HA@RFM@GP@SIN NPs (Fig. 9G and H). Notably, in the ovarian steroidogenesis pathway, HA@RFM@GP@SIN NPs restored progesterone and estriol levels to normal in the CIA group (Fig. 9I and J). Progesterone, as a novel anti-inflammatory hormone component, exhibits both non-specific and specific anti-inflammatory effects. The non-specific anti-inflammatory effect of progesterone involves the inhibition of NF-κB and cyclooxygenase, as well as a reduction in prostaglandin biosynthesis. Its specific anti-inflammatory mechanism is associated with the regulation of T-cell activation and the inhibition of proliferation pathways [42]. Estriol, the primary form of estrogen during pregnancy, has been found to stimulate IL-10 and suppress the secretion of TNF-α, thereby inducing a Th2 cell phenotype in T cells [43]. Studies have demonstrated that an elevated level of melatonin (1 mg/kg) in mice with CIA stimulates the immune system and increase the levels of IL-12 and nitric oxide, thus exacerbating RA. Accordingly, reducing melatonin levels has been considered as an effective treatment [44]. As indicated in Fig. 9K, melatonin expression was elevated in the CIA group, but reduced in both the normal and HA@RFM@GP@SIN groups. Hydroquinone, found in the tyrosine metabolic pathway, aggravates RA by activating the aryl hydrocarbon receptor and interleukin-17 pathways [45]. The hydroquinone content in the CIA group was heightened relative to the normal group; however, after treatment with HA@RFM@GP@SIN NPs, the hydroquinone content returned to normal levels (Fig. 9L). In conclusion, the treatment of RA with HA@RFM@GP@SIN NPs involves an increase in progesterone and estriol in the ovarian steroidogenesis pathway, the inhibition of melatonin in the tryptophan metabolism pathway, and the suppression of hydroquinone in the tyrosine metabolism pathway.

### Mechanisms underlying the efficacy of HA@RFM@GP@SIN NPs in arresting abnormal proliferation of RAFLS

**Fig. 10:**
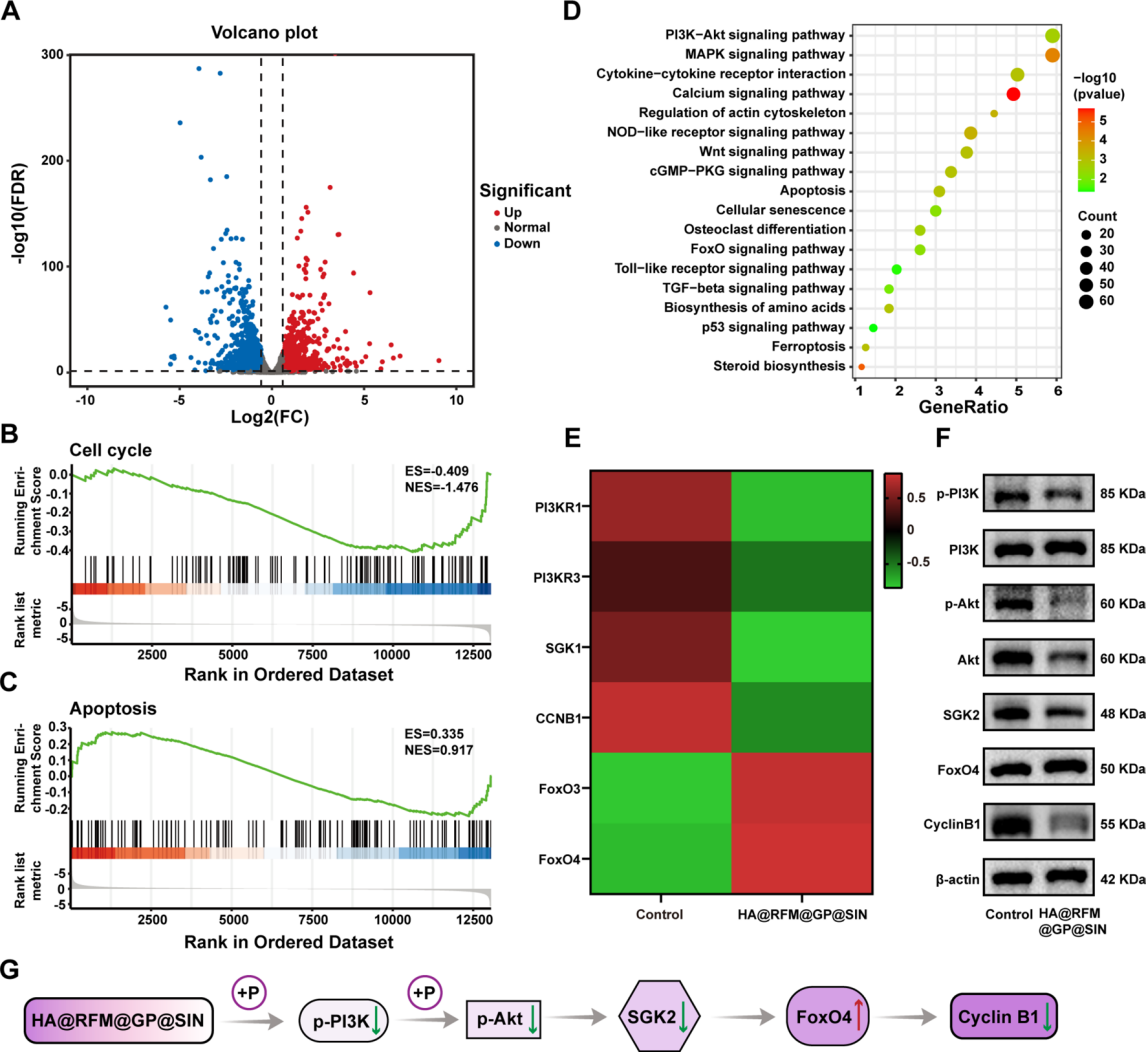
The molecular mechanisms of HA@RFM@GP@SIN NPs on RAFLS. (**A**) Volcano plot showing differential gene expression regulation from RNA-sequence analysis between the HA@RFM@GP@SIN and control groups. Red and green colors indicate upregulated and downregulated genes, respectively. (**B**) GSEA analysis of the cell cycle and (**C**) apoptosis after treatment with HA@RFM@GP@SIN NPs. (**D**) KEGG pathway analysis of differential genes between the HA@RFM@GP@SIN and control groups. (**E**) Heat map analysis of genes differentially expressed in RAFLS between the HA@RFM@GP@SIN and control groups. The color and intensity in each box represent changes in gene expression. Red represents up-regulated genes, and green represents downregulated genes. (**F**) WB assay of p-PI3K/PI3K, p-Akt/Akt, SGK2, FoxO4, Cyclin B1, and β-actin in RAFLS. (**G**) Mechanism diagram of HA@RFM@GP@SIN NPs to regulate cyclin B1 expression and arrest the cell cycle in the G2 phase by inhibiting the PI3K/Akt/SGK/FoxO pathway

Transcriptome analysis is a valuable tool for exploring the RNA expression levels of an organism in different states and uncovering the therapeutic mechanisms of drugs [46]. In our study, we investigated the mechanism by which HA@RFM@GP@SIN NPs inhibited the proliferation of RAFLS. Total RNA was collected from both the control and HA@RFM@GP@SIN groups, and subsequent analysis revealed 2061 differentially expressed genes between the two groups. Among these, upregulation of 988 genes while downregulation of 1073 genes, indicated significant differences in RNA expression levels between the HA@RFM@GP@SIN NPs and control groups. This is depicted in the volcano diagram (Fig. 10A), where upregulated genes are marked in red and downregulated genes in blue (fold change > 2 and *P* < 0.05). To gain a more comprehensive understanding of the overall changes in functional pathways and target key gene sets, we conducted gene set enrichment analysis (GSEA) [47]. The GSEA revealed the suppression of cell cycle pathway (Fig. 10B), while the induction of cell apoptosis pathway in the HA@RFM@GP@SIN NPs treatment group (Fig. 10C). These findings aligned with the earlier results obtained from cell cycle and apoptosis analyses (Fig. 4F and I). The KEGG pathway analysis uncovered that the differentially expressed genes were involved in various pathways that regulate cell proliferation, including the PI3K/Akt, MAPK, Wnt, p53, and FoxO pathways (Fig. 10D). Notably, the PI3K/Akt/SGK/FoxO pathway regulates the metabolic activity and inflammatory activation of RAFLS [48]. Heat map analysis indicated that HA@RFM@GP@SIN NPs reduced the expression of PI3K/Akt and SGK while increased the transcriptional activity of FoxO, leading to the inhibition of cyclin B1 (Fig. 10E). The results of western blot showed that HA@RFM@GP@SIN NPs suppressed the protein expression levels of p-PI3K/PI3K, p-Akt/Akt, SGK2, and cyclin B1, while upregulating FoxO4 in the RAFLS (Fig. 10F and Fig. S6). These findings demonstrated that HA@RFM@GP@SIN NPs hindered the proliferation of RAFLS by modulating the PI3K/Akt/SGK/FoxO pathway, resulting in reduced cyclin B1 expression and cell cycle arrest in the G2 phase (Fig. 10G).

### Safety evaluation of HA@RFM@GP@SIN NPs in vitro and in vivo

#### Biocompatibility evaluation of HA@RFM@GP@SIN NPs in vitro

**Fig. 11:**
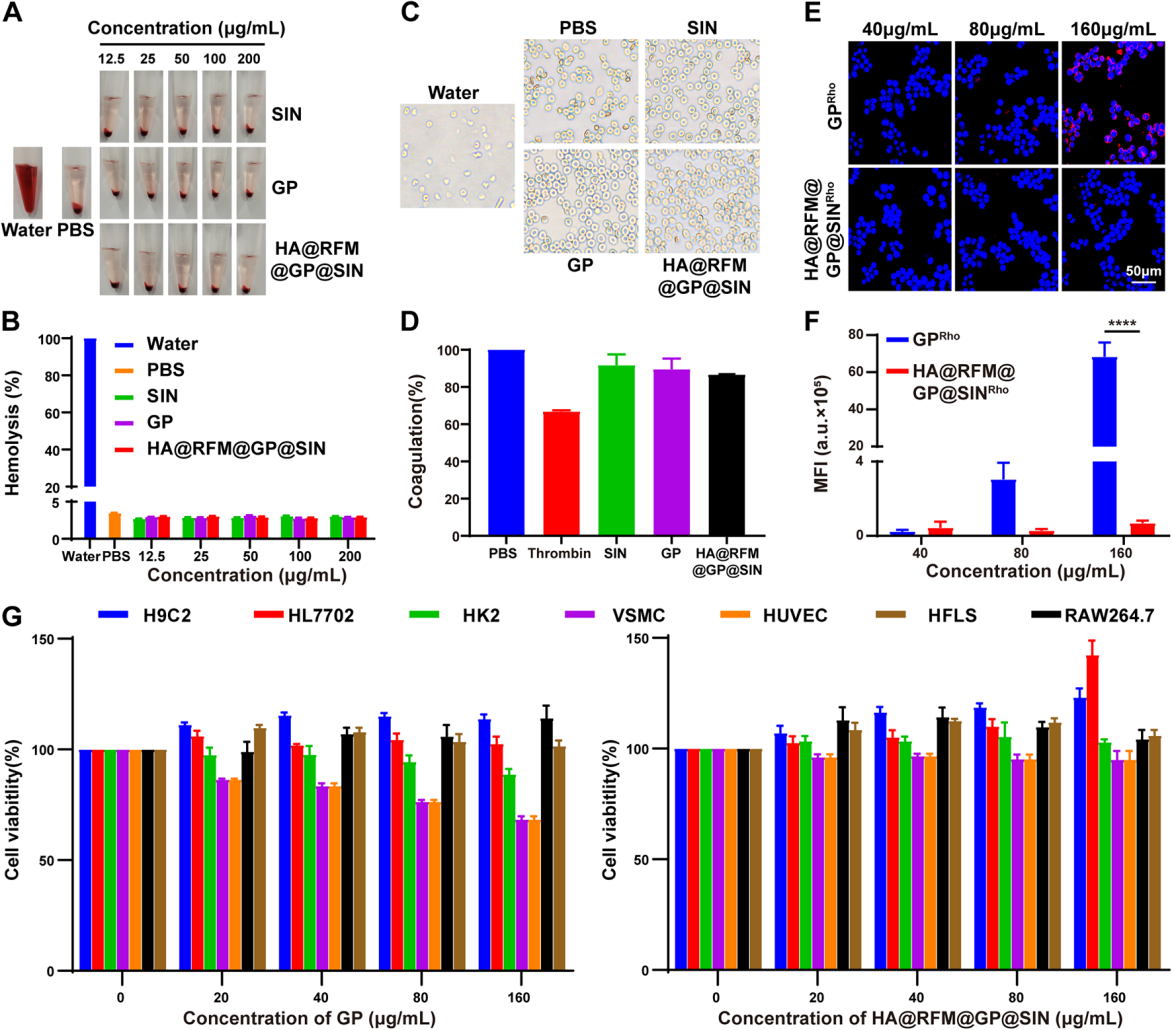
Biocompatibility evaluation of HA@RFM@GP@SIN NPs in vitro. (**A**) Images of hemolysis. (**B**) Quantitation of hemolysis rates. (**C**) Morphological images of erythrocytes incubated in water, PBS, SIN, GP NPs, or HA@RFM@GP@SIN NPs for 4 h. (**D**) Aggregation of platelets incubated with PBS, SIN, GP NPs, or HA@RFM@GP@SIN NPs for 4 h. (**E**) Fluorescence images and (**F**) quantitation of the immune evading ability of GP NPs or HA@RFM@GP NPs at 4 h. (**G**) The cell viability of H9C2, HL7702, HK2, VSMC, HUVEC, HFLS, and RAW264.7 cells treated with 0, 20, 40, 80, 160 µg/mL of GP NPs or HA@RFM@GP@SIN NPs for 24 h. Data are the means ± SEM, *n* = 3 per treatment, ^****^P < 0.0001

The safety of nanomaterials is crucial for their clinical applications. Firstly, the compatibility of HA@RFM@GP@SIN NPs was assessed by conducting hemolysis and platelet aggregation assays. Incubation of GP NPs or HA@RFM@GP@SIN NPs with different concentrations did not result in significant hemolysis (less than 5%) or morphological changes in rat erythrocytes (Fig. 11A-C). Moreover, a high dose of HA@RFM@GP@SIN NPs (200 µg/mL) exhibited minimal impact on platelet aggregation, indicating an extremely low risk of thrombosis (Fig. 11D). Additionally, the immune-escape ability of HA@RFM@GP@SIN NPs was examined in the mononuclear phagocyte system. When Rho-labeled nanomaterials were incubated with RAW264.7 cells for 4 h, GP NPs were progressively phagocytosed by RAW264.7 cells with increasing concentrations. However, HA@RFM@GP@SIN NPs were not engulfed by RAW264.7 cells under the same conditions (Fig. 11E and F). This result suggests that the RFM coating dampens macrophage-mediated phagocytosis due to the properties of the original cell membrane proteins. Several related cell lines were utilized to assess the cytotoxicity of HA@RFM@GP@SIN NPs (Fig. 11G). Both GP NPs and HA@RFM@GP@SIN NPs exhibited low cytotoxicity (over 80% activity) in H9C2 (heart), HL7702 (liver), HK2 (kidney), HFLS, and RAW264.7 cells. Additionally, GP NPs at concentrations of 80 and 160 µg/mL reduced VSMC and HUVEC cell viability, while HA@RFM@GP@SIN NPs did not affect their viability under the same conditions, demonstrating a protective effect on blood vessels. These findings indicate that HA@RFM@GP@SIN NPs are non-toxic to major organs such as the heart, liver, and kidney.

#### HA@RFM@GP@SIN NPs showed high safety in vivo

**Fig. 12:**
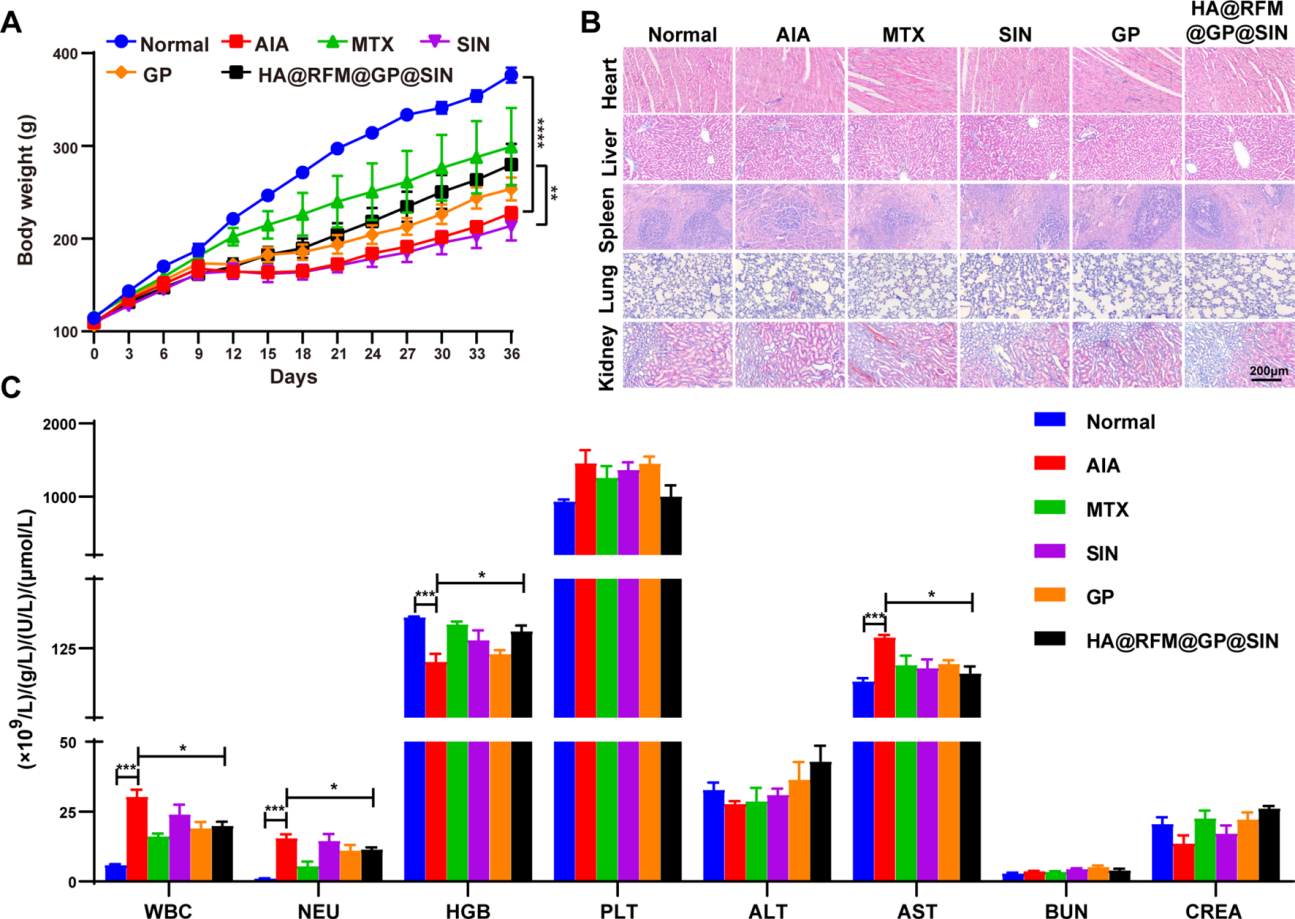
HA@RFM@GP@SIN NPs showed high safety in vivo. (**A**) The body weights of the rats in the normal, AIA, MTX, SIN, GP, and HA@RFM@GP@SIN groups. (**B**) H&E stained sections of heart, liver, spleen, lung, and kidney tissues from normal and AIA rats of different treatment groups. (**C**) Complete blood panel analysis of WBC, NEU, HGB, and PLT levels. Evaluation of hepatotoxicity and nephrotoxicity by measuring plasma levels of ALT, AST, BUN, and CREA. Data are the means ± SEM, *n* = 5 per treatment, ^*^P < 0.05, ^**^P < 0.01, ^***^P < 0.001, ^****^P < 0.0001

The in vivo relative toxicity of HA@RFM@GP@SIN NPs was further assessed by monitoring different parameters. Change in body weight serves as an important parameter for biosafety evaluation. As depicted in Fig. 12A, both the AIA and SIN groups of rats displayed significantly slower weight gain relative to the normal group. In contrast, the group treated with HA@RFM@GP@SIN NPs showed a greater elevation in body weight than those treated with SIN or GP NPs. H&E staining revealed hepatic lobular vein hyperplasia and blockage in the AIA and SIN groups. The spleen sections of the AIA group exhibited an indistinct boundary between the white and red pulp, with a significantly reduced proportion of red pulp. Alveolar epithelial cell edema, wall thickening, and cavity enlargement were observed in both the AIA and SIN groups. Furthermore, the renal tubules were dilated in the AIA and SIN groups, with irregularly shaped glomeruli in the AIA group. However, the HA@RFM@GP@SIN NPs groups displayed intact hepatic lobular venous lumen, well-defined and evenly distributed white and red pulp, slight alveolar wall thickening, and normal renal tubules and glomeruli (Fig. 12B). Moreover, routine blood tests exhibited a noteworthy elevation in white blood cell (WBC) and neutrophil granulocyte (NEU) levels within the AIA group in comparison to the normal group. Conversely, the HA@RFM@GP@SIN group exhibited decreased WBC and NEU levels (Fig. 12C). Hemoglobin (HGB) and platelet (PLT) levels in AIA rats were restored to normal following treatment with HA@RFM@GP@SIN NPs. Additionally, potential hepatotoxicity was evaluated by measuring liver enzymes such as alanine transaminase (ALT) and aspartate aminotransferase (AST), while potential nephrotoxicity was assessed using blood creatinine (CREA) and blood urea nitrogen (BUN) in rats. As illustrated in Fig. 12C, no significant disparities in ALT, BUN, and CREA levels were observed between the HA@RFM@GP@SIN NPs and normal groups. Furthermore, HA@RFM@GP@SIN NPs diminished AST levels in the AIA groups. These findings indicate that HA@RFM@GP@SIN NPs pose no hepatotoxic or nephrotoxic effects and might even enhance liver functionality. In conclusion, these outcomes provide evidence for the safety of HA@RFM@GP@SIN NPs in the treatment of RA.

## Conclusion

In this work, a biomimetic nanomedicine system named HA@RFM@GP@SIN NPs was successfully developed for the synergistic treatment of RA. In this nanomedicine system, GOQDs not only function as SIN carriers but also facilitate the transformation of macrophages from the M1 to M2 phenotype to exert anti-oxidant and anti-inflammatory effects, enabling collaborative therapy. Furthermore, this biomimetic nanomedicine system demonstrated precise targeting of the synovial lesion site in arthritis, collaborative regulation of synovial inflammation and hyperplasia, and efficient prevention of cartilage erosion and bone destruction. More notably, HA@RFM@GP@SIN NPs were demonstrated to exert anti-arthritic effects by intervening the PI3K/Akt/SGK/FoxO pathway, steroid hormone biosynthesis, ovarian steroidogenesis, tryptophan metabolism, and tyrosine metabolism. Additionally, both in vitro and in vivo assessments demonstrated favorable biocompatibility and biosafety of HA@RFM@GP@SIN NPs. In conclusion, this biomimetic nanomedicine system showcases the synergistic interplay between anti-proliferative and anti-inflammatory actions, emphasizing the significance of a collaborative therapeutic strategy for effective RA treatment.

### Electronic supplementary material

Below is the link to the electronic supplementary material.

Supplementary Material 1

## Data Availability

Data is provided within the manuscript or supplementary information files.
