# Supplementary material for: Graphene oxide quantum dots-loaded sinomenine hydrochloride nanocomplexes for effective treatment of rheumatoid arthritis via inducing macrophage repolarization and arresting abnormal proliferation of fibroblast-like synoviocytes
Source: J Nanobiotechnology. 2024 Jul 1;22:383. doi: 10.1186/s12951-024-02645-8 (PMC11218134; doi:10.1186/s12951-024-02645-8)
Supplement: Supplementary file 1 — Supplementary Material 1 [file 12951_2024_2645_MOESM1_ESM.docx]

**Supplementary data**

**Figure S1**

**
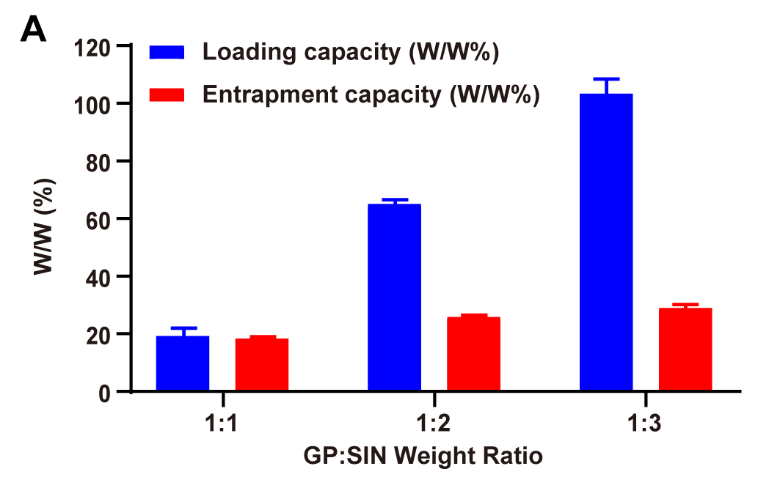
**

**Fig.S1. Fabrication of GP@SIN NPs.** (A) Encapsulation and loading efficiency of GP@SIN NPs at different mass ratios of GP NPs and SIN. Data are the means ± SEM, n = 3 per treatment.

**Figure S2**


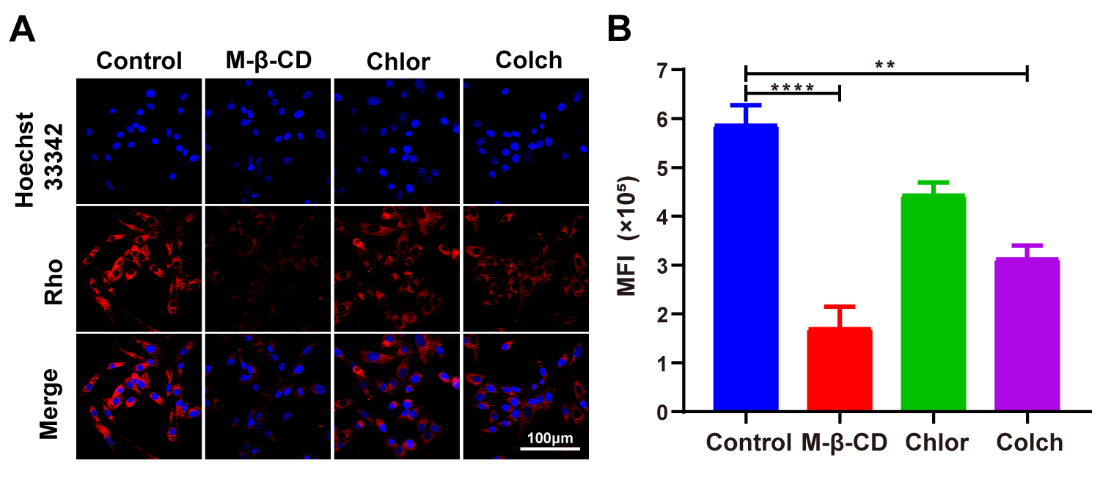


**Fig.S2. Uptake mechanism of HA@RFM@GP@SIN NPs.** (A) Representative fluorescent images and (B) MFI of the mechanism underlying the cellular uptake of HA@RFM@GP@SIN NPs by RAFLS (M-β-CD, caveolae-mediated endocytosis inhibitor; Chlor, clathrin-dependent endocytosis inhibitor; Colch, micropinocytosis inhibitor). Data are the means ± SEM, n = 3 per treatment, ^**^*P* < 0.01, ^****^*P* < 0.0001.

**Figure S3**


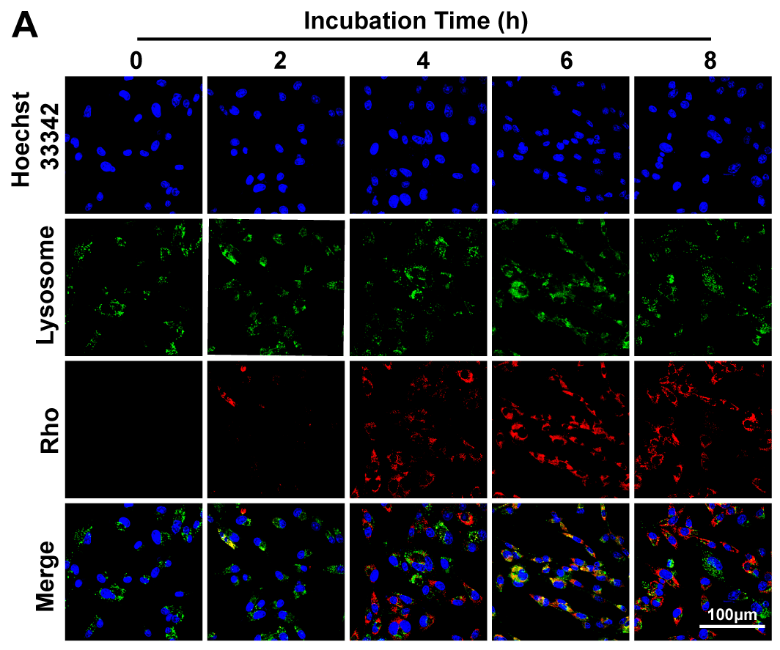


**Fig.S3. Intracellular localization of HA@RFM@GP@SIN NPs in RAFLS.** (A) CLSM images of the subcellular localization of HA@RFM@GP@SIN NPs in RAFLS at different time points.

**Figure S4**


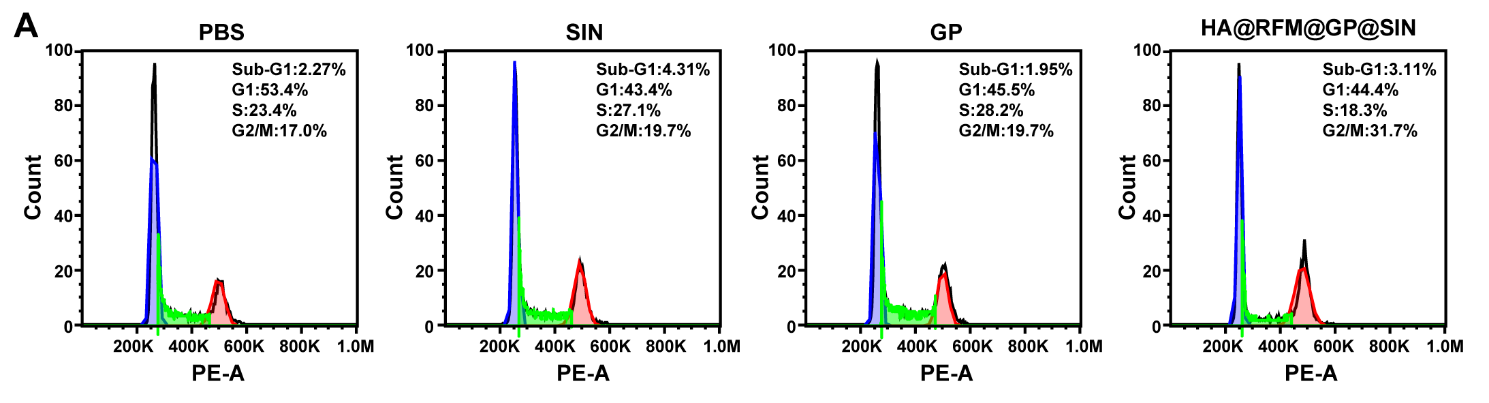


**Fig.S4. Cell cycle assay.** (A) Cell cycle distribution of RAFLS after with PBS, SIN, GP NPs, and HA@RFM@GP@SIN NPs treatment by flow cytometry.

**Figure S5**


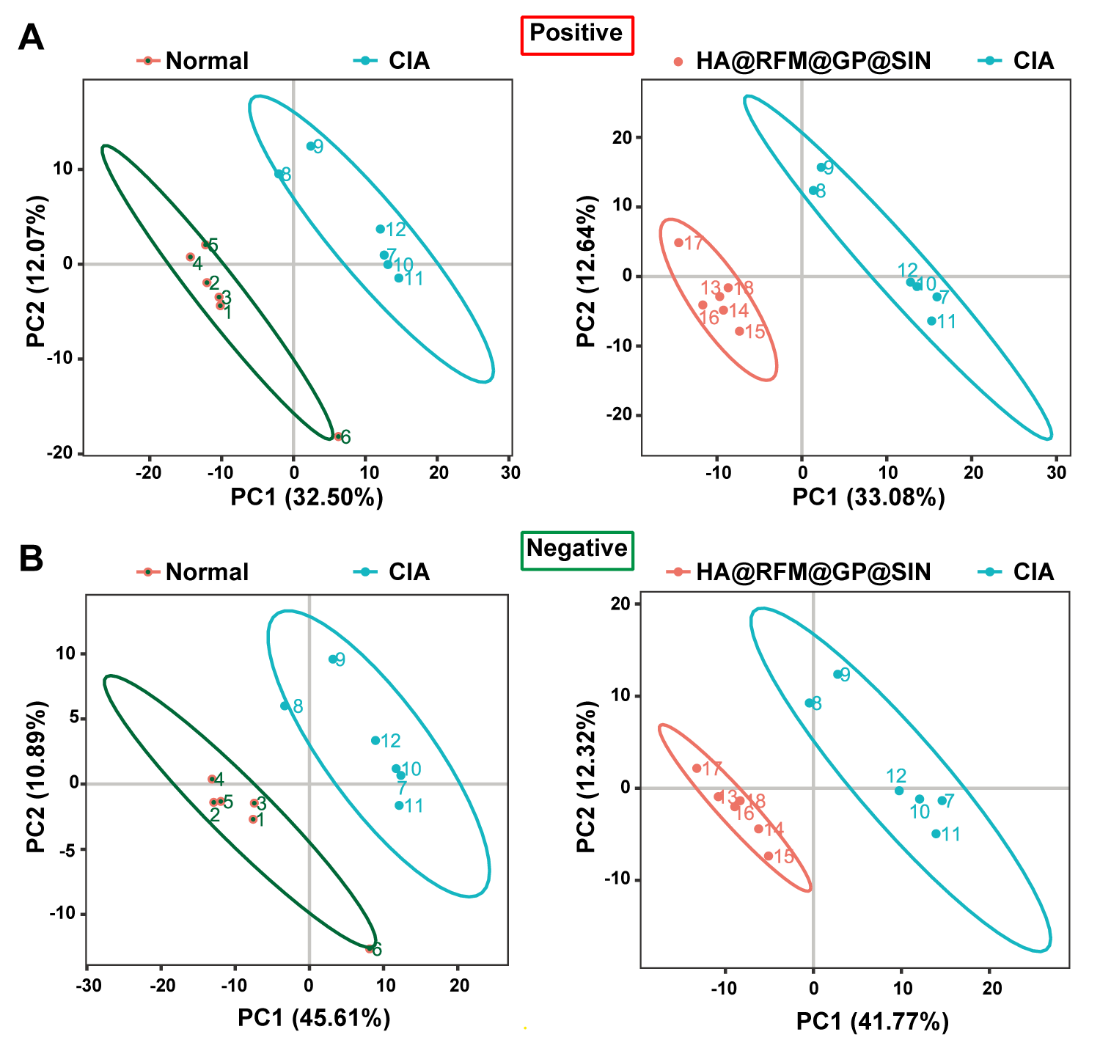


**Fig.S5. PLS-DA analysis of metabolomics.** (A) PLS-DA analysis of the normal, CIA, or HA@RFM@GP@SIN groups under positive and (B) negative ion modes.

**Figure S6**


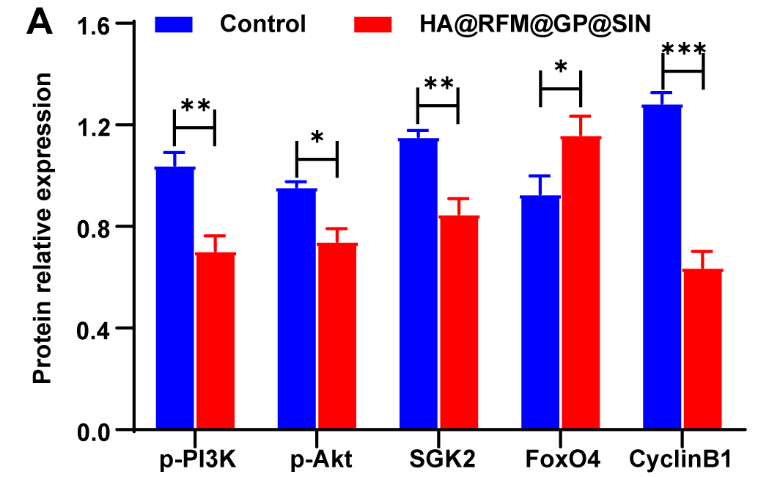


**Fig.S6. WB analysis of the molecular mechanisms of HA@RFM@GP@SIN NPs on RAFLS.** (A) Quantitative data of p-PI3K, p-Akt, SGK2, FoxO4 and CyclinB1, β-actin in RAFLS. Data are the means ± SEM, n = 3 per treatment, ^*^*P* < 0.05, ^**^*P* < 0.01, ^***^*P* < 0.001.
